# C/EBPβ-LIP induces cancer-type metabolic reprogramming by regulating the *let-7*/LIN28B circuit in mice

**DOI:** 10.1038/s42003-019-0461-z

**Published:** 2019-06-14

**Authors:** Tobias Ackermann, Götz Hartleben, Christine Müller, Guido Mastrobuoni, Marco Groth, Britt A. Sterken, Mohamad A. Zaini, Sameh A. Youssef, Hidde R. Zuidhof, Sara R. Krauss, Gertrud Kortman, Gerald de Haan, Alain de Bruin, Zhao-Qi Wang, Matthias Platzer, Stefan Kempa, Cornelis F. Calkhoven

**Affiliations:** 1 European Research Institute for the Biology of Ageing (ERIBA), University Medical Center Groningen, University of Groningen, 9700 AD Groningen, The Netherlands; 2 Leibniz Institute on Aging - Fritz Lipmann Institute, Beutenbergstrasse 11, D-07745 Jena, Germany; 3 Max Delbrück Center for Molecular Medicine, D-13092 Berlin, Germany; 4 Dutch Molecular Pathology Centre, Faculty of Veterinary Medicine, Utrecht University, Yalelaan 1, NL-3584 CL Utrecht, the Netherlands

**Keywords:** Cell growth, Cancer metabolism

## Abstract

The transcription factors LAP1, LAP2 and LIP are derived from the *Cebpb*-mRNA through the use of alternative start codons. High LIP expression has been associated with human cancer and increased cancer incidence in mice. However, how LIP contributes to cellular transformation is poorly understood. Here we present that LIP induces aerobic glycolysis and mitochondrial respiration reminiscent of cancer metabolism. We show that LIP-induced metabolic programming is dependent on the RNA-binding protein LIN28B, a translational regulator of glycolytic and mitochondrial enzymes with known oncogenic function. LIP activates LIN28B through repression of the *let-7* microRNA family that targets the *Lin28b*-mRNA. Transgenic mice overexpressing LIP have reduced levels of *let-7* and increased LIN28B expression, which is associated with metabolic reprogramming as shown in primary bone marrow cells, and with hyperplasia in the skin. This study establishes LIP as an inducer of cancer-type metabolic reprogramming and as a regulator of the *let-7*/LIN28B regulatory circuit.

## Abstract (web summary)

Tobias Ackermann et al. show that the C/EBPβ Liver-enriched inhibitory protein (LIP) induces aerobic glycolysis and mitochondrial respiration by suppressing the *let-7* microRNA family that targets the *Lin28b*-mRNA. This study identifies LIP as a regulator of metabolic reprogramming that is reminiscent of cancer metabolism.

## Introduction

Proliferating (cancer) cells generate a diversity of carbon intermediates and reducing power in the form of NADPH, needed for biosynthetic reactions, by markedly increasing glucose uptake and glucose catabolism. During this process excess of the cytosolic end product of glycolysis, pyruvate, is converted into lactate and secreted into the extracellular environment (the so-called Warburg effect)^1,2^. In addition, cancer cells increase mitochondrial activity and boost both the replenishment (anaplerosis) and the clearance (cataplerosis) of intermediates of the TCA cycle. Thereby, the mitochondria serve as a metabolic hub that produces biomolecule intermediates in addition to energy^3^.

The metabolic reprogramming of cancer cells is induced by deregulation or mutations in metabolic enzymes, components of signalling pathways and gene regulatory factors. LIN28A and its paralogue LIN28B are such key metabolic reprogramming factors. As RNA-binding proteins, LIN28A/B stimulate the translation of several glycolytic and mitochondrial enzymes in the context of cell metabolism, tissue repair and cancer^4–7^. *Lin28a/b* are highly expressed during embryogenesis but silent in differentiated cells of adult tissues^8^. *Lin28b* is aberrantly high expressed in various tumour types^9,10^ and transgenic overexpression is sufficient to drive cancer and is required for tumour maintenance^4,11–13^. LIN28A/B repress the maturation of the *let-7* family of microRNAs consisting of nine (*a, b, c, d, e, f, g, i* and *miR-98*) functionally redundant microRNAs that originate from eight conserved *let-7* microRNA expressing clusters in humans and mice^14–18^. The *let-7* microRNAs function as tumour suppressors by inhibiting the mRNAs of various oncogenes and cell cycle regulators, including *Lin28a/b*^19,20^. Thus, *let-7* and *Lin28a/b* have reciprocal functions in a regulatory circuit in which *let-7* represses *Lin28a/b*-mRNAs, while LIN28A/B represses *let-7* maturation^20^.

When addressing the functions of the transcription factor CCAAT/Enhancer Binding Protein beta (C/EBPβ), it should always be considered that translation of the Cebpb-mRNA gives rise to three protein isoforms with different functions^21–24^. The isoforms called LAP1 and LAP2 (Liver-enriched transcriptional activating protein) are complete transcriptional activators, while the N-terminally truncated isoform LIP (Liver-enriched inhibitory protein) lacks transactivation domains and acts by inhibiting the functions of LAP and other C/EBPs through competitive binding to the same DNA-recognition sites. Expression of LIP is regulated by a dedicated translation control mechanism that requires a *cis*-regulatory upstream open reading frame (uORF) in the Cebpb-mRNA leader sequence (Supplementary Fig. 1a)^22–24^.

High expression of LIP is associated with breast cancer, ovarian cancer and anaplastic large cell lymphoma^25–32^, and cellular transformation in cell culture^22^. Furthermore, knockin mice that either express mono- or bi-allelic LIP-only display enhanced tumourigenesis^33^. Together, the studies demonstrate that LIP has oncogenic capacities, but whether and how metabolic functions of LIP are involved is not known.

Here we show that LIP enhances aerobic glycolysis and mitochondrial respiration, resembling cancer cell metabolism. Using genome wide transcriptome and whole cell proteome analysis we identify LIN28B as a required mediator of LIP-controlled metabolic regulation. We provide evidence that LIP controls *Lin28b* expression though transcriptional repression of *let-7*. Furthermore, analysis of a mouse model with LIP overexpression confirms the LIP-*let-7-Lin28b* regulation in vivo, which is associated with metabolic reprogramming in primary bone marrow cells and an increase in immature cells as well as hyperplasia in the skin. Our data suggest an important role of LIP in controlling the let-7/LIN28B regulatory circuitry and thereby regulating cellular metabolism and possibly inducing a tumour prone state.

## Results

### LIP enhances aerobic glycolysis and mitochondrial metabolism

**Fig. 1:**
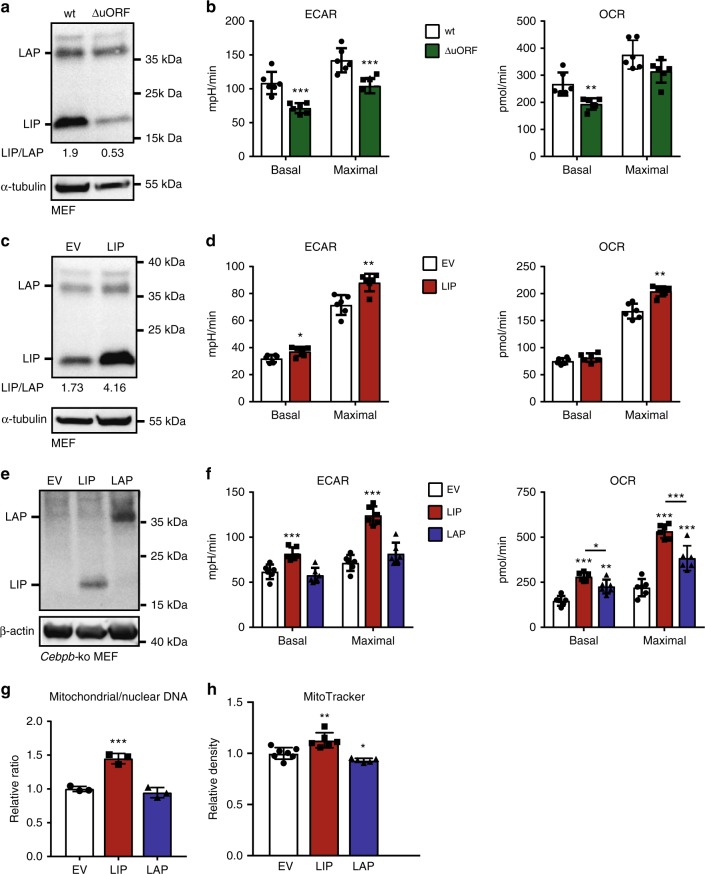
C/EBPβ-LAP and -LIP isoforms regulate cellular metabolism. **a** Immunoblot analysis of C/EBPβ-LAP and C/EBPβ-LIP expression in mouse embryonic fibroblasts (MEFs) derived from wt or C/EBPβ^ΔuORF^ mice. α-tubulin is used for loading control. Uncropped images are shown in Supplementary Fig. 7. **b** Extracellular acidification rate (ECAR) and oxygen consumption rate (OCR) of MEFs derived from wt or C/EBPβ^ΔuORF^ mice (*n* = 6). **c** Immunoblot analysis of C/EBPβ-LAP and C/EBPβ-LIP expression in wt MEFs and with ectopic expression of LIP. α-tubulin is used for loading control. Uncropped images are shown in Supplementary Fig. 7. **d** ECAR and OCR of wt MEFs with control empty vector (EV) or ectopic expression of LIP (*n* = 6). **e** Immunoblot analysis of C/EBPβ-LAP and C/EBPβ-LIP expression in *Cebpb*-knockout (ko) MEFs with control empty vector (EV) or ectopic expression of LIP or LAP. β-actin is used for loading control. Uncropped images are shown in Supplementary Fig. 7. **f** ECAR and OCR of *Cebpb*-ko MEFs with control empty vector (EV) or ectopic expression of LIP or LAP (*n* = 6). **g** Mitochondrial/nuclear DNA ratio and **h** MitoTracker quantification of *Cebpb*-ko MEFs with control empty vector (EV) or ectopic expression of LIP or LAP (*n* = 7 for EV, *n* = 6 for LIP and *n* = 5 for LAP). Statistical differences were analysed by Student’s *t*-tests. Error bars represent SD, *P < 0.05, **P < 0.01, ***P < 0. 001

In the context of earlier studies, we repeatedly observed that cellular overexpression of LIP but not of LAP results in rapid acidification of the cell culture medium. Therefore, we investigated a possible involvement of LIP and LAP in the regulation of cellular metabolism. To examine LIP-dependent cellular metabolism we measured the extracellular acidification rate (ECAR) as an indicator for glycolytic flux and the oxygen consumption rate (OCR) as a measure for mitochondrial respiration using the Seahorse XF96 analyser in wild-type (wt) mouse embryonic fibroblasts (MEFs) versus MEFs derived from C/EBPβ^ΔuORF^ mice that express lower LIP/LAP ratios compared to wt due to deficient endogenous LIP production^23,24,34^ (Fig. 1a and Supplementary Fig. 1a). Basal ECAR, maximal ECAR (treatment with ATP synthase inhibitor oligomycin) and basal OCR, but not maximal OCR (treatment with mitochondrial uncoupler 2,4-dinitrophenol, DNP), were decreased in C/EBPβ^ΔuORF^ MEFs compared to wt MEFs (Fig. 1b). Conversely, ectopic overexpression of LIP in wt MEFs shifting C/EBPβ expression to higher LIP/LAP ratios (Fig. 1c) resulted in an increase in basal and maximal ECAR as well as an increase in maximal OCR (Fig. 1d). To investigate the function of individual C/EBPβ isoforms we separately overexpressed LAP or LIP in immortalized *Cebpb*-knockout (ko) MEFs (Fig. 1e). LIP expression was sufficient to induce both higher basal and maximal ECAR and OCR, while LAP expression resulted only in higher OCRs, albeit not as strong as LIP (Fig. 1f). Furthermore, expression of LIP in *Cebpb*-ko MEFs increased both the ratio of mitochondrial to genomic DNA (Fig. 1g) and mitochondrial mass as detected by staining with MitoTracker (Fig. 1h and Supplementary Fig. 1b), while expression of LAP did not result in noticeable changes. Overexpression of LIP also increased basal and maximal ECAR and OCR in the human hepatocellular carcinoma cell line Hepa1-6 and in the breast cancer cell lines BT20 and T47D (Supplementary Fig. 1c–h). Thus, LIP alone or a high LIP/LAP ratio enhances the cellular metabolic rate with an increase in both aerobic glycolysis and mitochondrial respiration capacity, which is reminiscent of cancer cell metabolism.

**Fig. 2:**
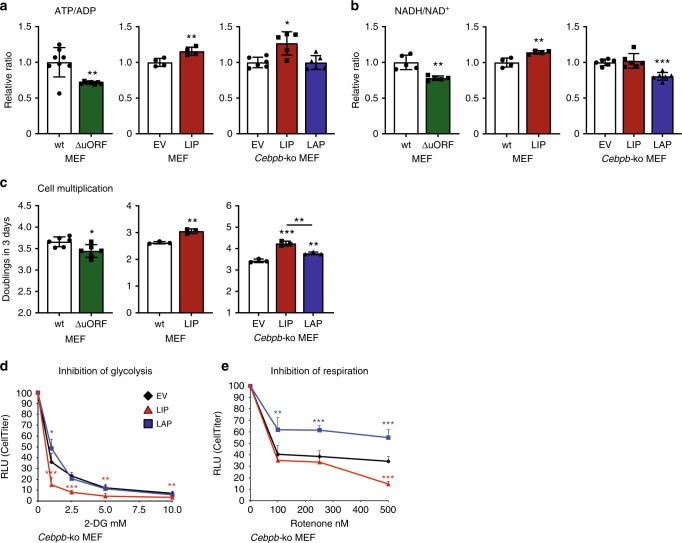
C/EBPβ-LAP and -LIP isoforms regulate cellular proliferation. **a** Relative ATP/ADP ratio in MEFs derived from, wt or C/EBPβ^ΔuORF^ mice (*n* = 8), wt MEFs with control empty vector (EV) or ectopic expression of LIP (*n* = 4), and *Cebpb*-ko MEFs with control empty vector (EV) or ectopic expression of LIP or LAP (*n* = 6). **b** Relative NADH/NAD^+^ ratio in, wt or C/EBPβ^ΔuORF^ MEFs (*n* = 6), wt MEFs with control empty vector (EV) or ectopic expression of LIP (*n* = 6), and *Cebpb*-ko MEFs with control empty vector (EV) or ectopic expression of LIP or LAP (*n* = 6). **c** Cell population doublings after three days of cell culture of, wt or C/EBPβ^ΔuORF^ MEFs (*n* = 6), wt MEFs with control empty vector (EV) or ectopic expression of LIP (*n* = 3), and *Cebpb*-ko MEFs with control empty vector (EV) or ectopic expression of LIP or LAP (*n* = 3). **d** Dose-response curve of inhibition of glycolysis by 2-DG in *Cebpb*-ko MEFs with control empty vector (EV) or ectopic expression of LIP or LAP (*n* = 6). **e** Dose-response curve of inhibition of mitochondrial respiration by rotenone in *Cebpb*-ko MEFs with control empty vector (EV) or ectopic expression of LIP or LAP (*n* = 6). Statistical differences were analysed by Student’s *t*-tests. Error bars represent SD, *P < 0.05, **P < 0.01, ***P < 0. 001

Proliferating cells, including cancer cells, adjust metabolism to maintain high ratios of ATP/ADP and NADH/NAD^+^ that are required for cell growth, proliferation and survival^2^. LIP-deficient C/EBPβ^ΔuORF^ MEFs maintained lower ATP/ADP ratios compared to wt cells, while ectopic expression of LIP in either wt or C/EBPβ-deficient MEFs resulted in an increase in the ATP/ADP ratio (Fig. 2a). Similarly, the LIP-deficient C/EBPβ^ΔuORF^ MEFs maintained lower NADH/NAD^+^ ratios, while ectopic expression of LIP in wt MEFs increased the NADH/NAD^+^ ratio. In C/EBPβ-deficient MEFs LIP had no effect on the NADH/NAD^+^ ratio while LAP reduced the NADH/NAD^+^ ratio, indicating that LIP indirectly regulates the NADH/NAD^+^ ratio in wt and C/EBPβ^ΔuORF^ MEFs by inhibiting the function of LAP (Fig. 2b). Furthermore, the cell proliferation rate was reduced for the LIP-deficient C/EBPβ^ΔuORF^ MEFs compared to wt MEFs, while ectopic expression of LIP in wt MEFs stimulated proliferation (Fig. 2c). Finally, ectopic expression of LIP in *Cebpb*-ko MEFs more strongly stimulated proliferation than ectopic expression of LAP (Fig. 2c). To address whether LIP-induced cell proliferation critically depends on glycolysis or mitochondrial respiration we performed dose-response experiments using either the glycolytic inhibitor 2-deoxyglucose (2-DG) or the mitochondrial complex 1 inhibitor rotenone. Cells expressing LIP were more sensitive to 2-DG compared to LAP expressing cells or control cells that have similar dose response curves (Fig. 2d). In addition, LIP expressing cells were more sensitive to rotenone compared to control cells while LAP expressing cells showed a strongly reduced sensitivity to rotenone (Fig. 2e).

Taken together, these data suggest that LIP induces a proliferation-supporting metabolic shift toward enhanced aerobic glycolysis and mitochondrial respiration and that LIP-expressing cells depend on these metabolic alterations for enhanced proliferation.

### Regulation by LIP involves post-transcriptional mechanism

**Fig. 3:**
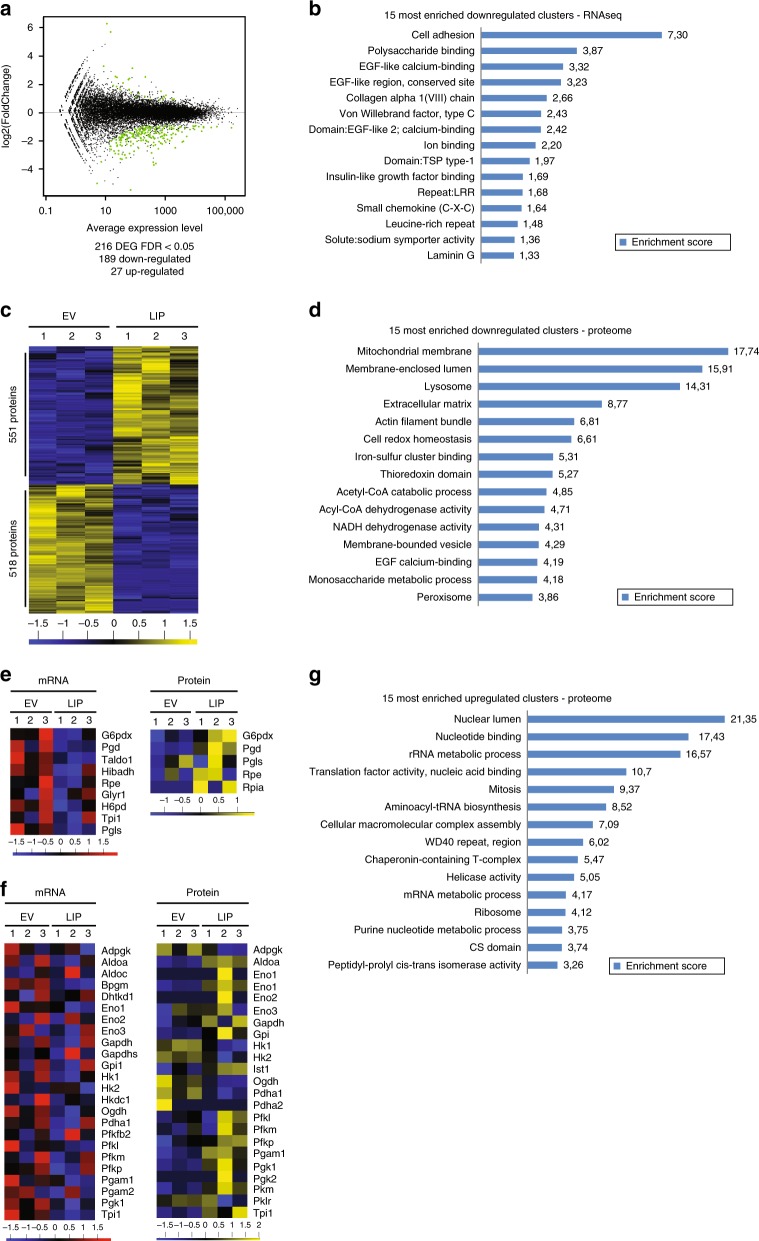
LIP differentially regulates transcriptome and proteome. **a** Differentially expressed genes (DEG) by ectopic expression of LIP in *Cebpb*-ko MEFs compared to control empty vector (EV) with an FDR < 0.05. **b** 15 most enriched functional clusters (DAVID) of mRNAs downregulated in LIP expressing *Cebpb*-ko MEFs. **c** Heat map representation of calculated z-scores for differentially expressed proteins in response to LIP expression in *Cebpb*-ko MEFs compared to EV control. **d** 15 most enriched functional clusters (DAVID) of proteins downregulated in LIP expressing *Cebpb*-ko MEFs. **e** Heat map representation of calculated z-scores for mRNAs and proteins of the pentose-phosphate-shuttle pathway. **f** Heat map representation of calculated z-scores for mRNAs and proteins of the glycolysis pathway. **g** 15 most enriched functional clusters (DAVID) of proteins upregulated in LIP expressing *Cebpb*-ko MEFs. For panels c, e and f z-scores as the number of standard deviations from the mean are depicted below the heat maps; increased colour intensity (+red/-green for mRNA and +yellow/-blue for protein) represents expression levels that are higher or lower than the mean levels from both conditions relative to the standard deviation associated by the mean

LIP is considered to mainly function by inhibiting transcriptional activities of LAP and probably of other C/EBP members through competitive binding to the same DNA-recognition sites. Although the enhanced metabolism induced by LIP might be explained by transcriptional upregulation of involved metabolic genes, the question was if and how LIP as a designated transcriptional repressor could be involved in such a transcriptional upregulation. To solve this question we studied changes in transcriptome and proteome induced by LAP or LIP using *Cebpb*-ko MEFs that express either LAP or LIP compared to C/EBPβ-deficient empty vector control cells. Surprisingly, LAP overexpression only changed the expression of 11 transcripts compared to the C/EBPβ-deficient empty vector control (Supplementary Fig. 2a). The overexpression of LIP resulted in the down-regulation of 189 genes and up-regulation of 27 genes; confirming that LIP mainly functions as a transcriptional inhibitor (Fig. 3a). Function clustering analysis of the downregulated genes using DAVID (https://david-d.ncifcrf.gov/) revealed that the three highest enriched clusters consist of genes associated with extracellular matrix, cell adhesion and collagen subtypes (Fig. 3b). These genes are involved in cell-cell interactions and their downregulation might result in an increased migration and invasion capacity of high LIP expressing cells^35,36^. However, notwithstanding its potential importance for oncogenic activities of LIP the transcriptome data do not explain the metabolic phenotypes induced by LIP.

In agreement with the results of the transcriptome, the overexpression of LAP had limited effects on the proteome of the cell. A statistical analysis did not identify significantly regulated proteins between C/EBPβ-deficient and LAP overexpressing cells (Supplementary Fig. 2b). On the contrary, overexpression of LIP resulted in marked changes in the cellular proteome with an increase in the expression of 551 proteins and a decrease in 518 proteins (Fig. 3c). Similar to the transcriptome analysis, functional clustering analysis (DAVID) of the downregulated proteins upon LIP expression showed an enrichment for proteins with function in cell-cell interactions. Furthermore, proteins belonging to catabolic organelles (peroxisome, lysosome and the mitochondrial membrane) and catabolic pathways (acyl-CoA dehydrogenase activity (fatty acid catabolism and acetyl-CoA catabolic process) were found downregulated upon LIP overexpression (Fig. 3d). In order to address the observed LIP-dependent increase in glycolysis we compared the mRNA and protein levels of glycolytic enzymes and enzymes of the pentose phosphate pathway by filtering our omics data with the Gene Ontology Biology Process (GOBP) annotations. Although LIP does not significantly alter relevant mRNA levels in both pathways, the protein levels of several enzymes of the glycolytic (Fig. 3e and supplementary Table 1) and pentose phosphate (Fig. 3f and supplementary Table 1) pathways are elevated in LIP expressing cells. In addition, proteins upregulated by LIP cluster in rRNA and mRNA metabolic processes, translation initiation and ribosomes as well as in proteins involved in purine biosynthesis and mitosis (DAVID) (Fig. 3g). Overall the observed LIP-induced changes in the proteome are associated with cell growth and proliferation. The discrepancy between the alterations for metabolic mRNAs and proteins as well as the general imbalance between the number of mRNAs and proteins (27 mRNAs vs. 551 proteins) that are upregulated by LIP indicate that LIP induces post-transcriptional mechanisms to control cellular metabolism and proliferation.

### LIN28B is required for LIP-induced metabolic reprogramming

**Fig. 4:**
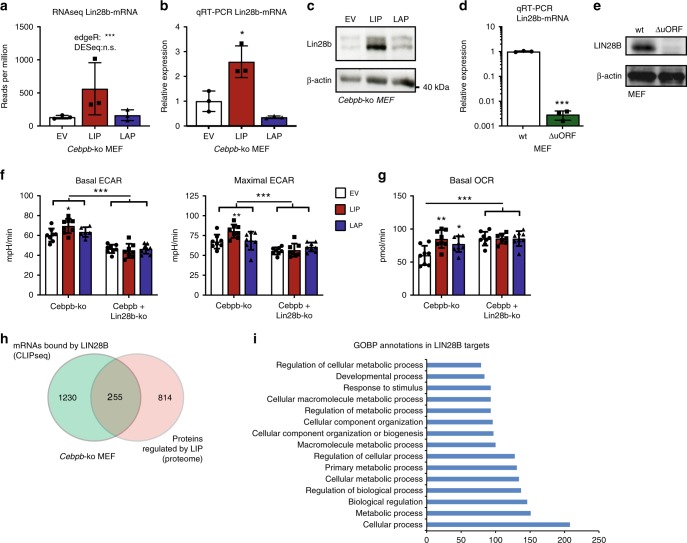
LIP requires *Lin28b* to regulate cellular metabolism. **a** *Lin28b* RNA sequencing reads in LIP or LAP expressing *Cebpb*-ko MEFs compared to control empty vector (EV) (*n* = 3). **b** Relative *Lin28b*-mRNA expression levels by qRT-PCR (*n* = 3) and **c** immunoblot analysis of LIN28B protein expression in LIP or LAP expressing *Cebpb*-ko MEFs compared to control empty vector (EV). β-actin is used for loading control. Uncropped images are shown in Supplementary Fig. 7. **d** Relative *Lin28b*-mRNA expression levels by qRT-PCR (*n* = 3). **e** Immunoblot analysis of LIN28B protein expression in wt MEFs compared to LIP-deficient C/EBPβ^ΔuORF^ MEFs. β-actin is used for loading control. Uncropped images are shown in Supplementary Fig. 7. **f** Basal and maximal ECAR of, *Cebpb*-ko MEFs with control empty vector (EV) or ectopic expression of LIP or LAP or with additional *Lin28b*-knockout (*Lin28b*-ko) (*n* = 6). **g** Basal OCR of *Cebpb*-ko MEFs with control empty vector (EV) or with ectopic expression of LIP or LAP, or with additional *Lin28b*-ko (*n* = 6). **h** Venn diagram showing overlap between LIP-regulated proteins (proteome, this study) and LIN28B targets (CLIPseq^4^). **i** GOBP annotations of LIN28B targets that are differential expressed in the LIP proteome. Statistical differences were analysed by Student’s *t*-tests. Error bars represent ±SD, *P < 0.05, **P < 0.01, ***P < 0.001

In the search for regulators of cell metabolism that use post-transcriptional mechanisms and are regulated by C/EBPβ, we re-analysed the transcriptome data. We found the Lin28b-transcript to be upregulated by LIP overexpression in C/EBPβ-deficient MEFs (Fig. 4a). This was confirmed by quantitative real-time PCR (qRT-PCR) (Fig. 4b) and immunoblot analysis (Fig. 4c), showing that both Lin28b/LIN28B mRNA and protein levels are strongly induced by LIP but not by LAP. Moreover, we found strongly reduced Lin28b-mRNA and LIN28B protein expression in the C/EBPβ^ΔuORF^ MEFs that are deficient in LIP (Fig. 4d, e). In addition, ectopic LIP expression in wt MEFs, Hepa1-6 or T47D cells resulted in elevated Lin28b-mRNA expression (Supplementary Fig. 3a–c). Thus, our data show that LIP but not LAP induces LIN28B expression, a protein known to regulate the translation of several glycolytic and mitochondrial enzymes in order to increase cellular metabolism and energy production^5,6^. We did not detect sequence reads for the Lin28b-paralogue Lin28a in the transcriptome. In addition, the LIN28A protein was not detected in the MEFs by immunoblot analysis (Supplementary Fig. 4a). Note, however, that in T47D cells LIN28A is expressed and that LIP overexpression causes an increase in LIN28A.

To investigate the requirement of LIN28B for LIP-mediated metabolic alterations we generated a *Lin28b*-knockout by CRISPR/Cas9 genomic editing using the C/EBPβ-deficient MEFs to create *C/ebpβ/Lin28b* double-knockout MEFs (Supplementary Fig. 3e). All effects of ectopic expression of LIP or LAP on OCR and ECAR in MEFs were abrogated by *Lin28b*-deficiency (Fig. 4f, g and Supplementary Fig. 3f, g, h). Hence, these data show that LIP induces *Lin28b* expression and that LIN28B is required for LIP-mediated regulation of cell metabolism.

Next, we compared known LIN28B-bound RNAs from published CLIP-Seq (cross-linking immunoprecipitation coupled with high-throughput sequencing) data^4^ with the LIP-regulated proteome for overlapping targets. This revealed that 255 of the 1069 differentially expressed proteins in LIP expressing cells are translated from mRNAs that are bound by LIN28B (Fig. 4h). Gene ontology biology process (GOBP) analysis showed that 150 of these 255 proteins are involved in metabolic processes (GO:0008152) (Fig. 4i). In contrast, only 22 mRNAs that are differentially expressed in LIP expressing cells, are LIN28B targets corroborating the post-transcriptional nature of the LIP-LIN28B regulatory effects (Supplementary Fig. 3d).

Taken together, our data show that LIN28B-regulated proteins are upregulated by LIP and that the LIN28B function is involved in LIP-controlled regulation of metabolism.

### Regulation of *Lin28b* by LIP involves *let-7* microRNAs

**Fig. 5:**
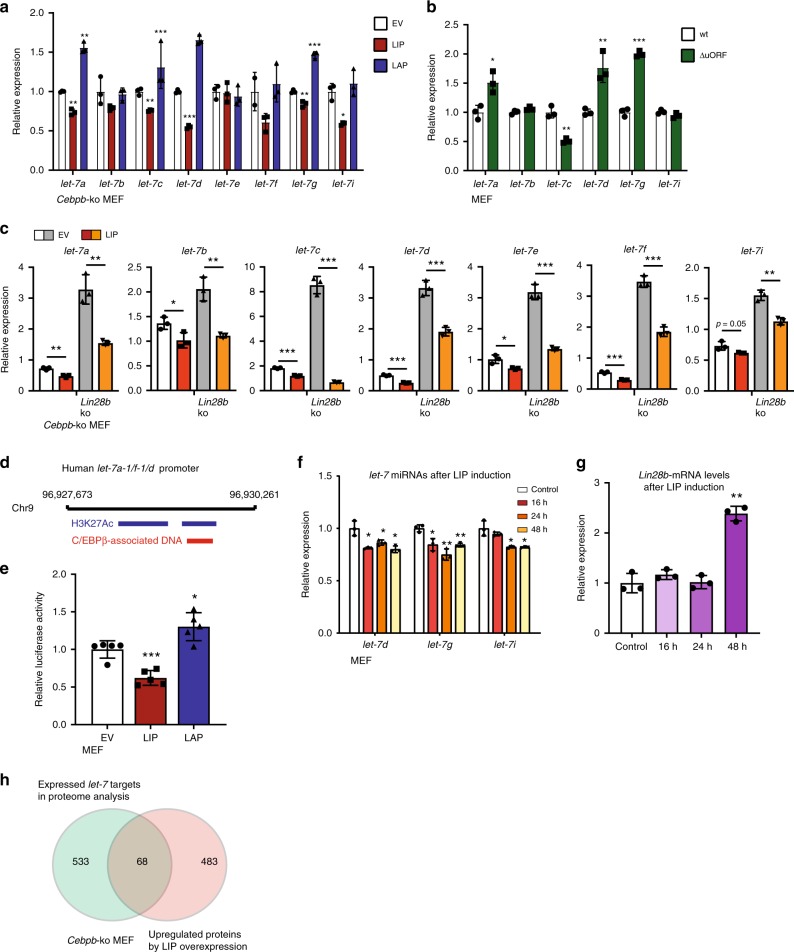
LIP and LAP transcriptionally regulate *let-7* microRNAs. **a** Expression levels of *let-7a, b, c, d, e, f, g* and *i* in *Cebpb*-ko MEFs with control empty vector (EV) or with ectopic expression of LIP or LAP (*n* = 3). **b** Expression levels of *let-7a, b, c, d, g* and *i* in wt or C/EBPβ^ΔuORF^ MEFs (*n* = 3). **c** Expression levels of *let-7a, b, c, d, e, f* and *i* in *Cebpb*-ko MEFs or *Cebpb/Lin28b*-dko MEFs with control empty vector (EV) or with ectopic expression of LIP. **d** A schematic visualization of the *let-7a-1/f-1/d* promoter with H3K27 acetylation and C/EBPβ-associated DNA fragments (http://genome.ucsc.edu/ENCODE/). **e** Bar graph of *let-7a-1/f-1/d* promoter-reporter activity in response to LIP or LAP expression in MEFs compared to EV control (*n* = 5). **f** Bar graph of expression levels of *let-7d, g* and *i* in wt MEFs and 16, 24 and 48 h after LIP induction (*n* = 3), and **g** Bar graph of the corresponding *Lin28b*-mRNA expression levels (*n* = 3). **h** Venn diagram showing overlap between proteins upregulated by LIP and *let-7* targets in the proteome analysis. Statistical differences were analysed by Student’s *t*-tests. Error bars represent +SD, *P < 0.05, **P < 0.01, ***P < 0.001

The fact that Lin28b-mRNA is upregulated by the transcriptional *repressor* LIP suggests the involvement of an intermediate repressor and primed us to investigate the microRNA family *let-7*, which is known to downregulate Lin28b-mRNA. Overexpression of LIP in C/EBPβ-deficient MEFs led to a down regulation of most of the eight *let-7* family members (Fig. 5a), while overexpression of LAP resulted in increased levels *of let-7a, d* and *g* (Fig. 5a). Moreover, in the LIP-deficient C/EBPβ^ΔuORF^ fibroblasts the levels of *let-7a*, *d* and *g* were found increased (*let-7c* is decreased) (Fig. 5b). Since LIN28B inhibits *let-7* maturation we examined a potential involvement of Lin28B in the LIP induced repression of the *let-7* microRNAs. Knockout of *lin28b* resulted in the expected upregulation of *let-7* microRNAs (*a, b, c, d, e, f* and *i*) from the different clusters (Fig. 5c). Ectopic expression of LIP in these *lin28b*-ko cells suppressed the elevated *let-7* microRNAs in all tested cases (Fig. 5c). This suggests that LIP can suppress *let-7* expression independently of LIN28B. Furthermore, ectopic overexpression of LIP in human breast cancer cell lines T47D and MCF7 led to a decrease in different *let-7* microRNAs, albeit with differences between the cell types (Supplementary Fig. 4b, c). Taken together, these data indicate that *let-7* microRNAs are differentially regulated by C/EBPβ; the transcriptional inhibitor LIP decreases *let-7* and the transcriptional activator LAP increases *let-7*.

The ENCODE database (http://genome.ucsc.edu/ENCODE/) lists C/EBPβ-associated DNA fragments with proximity to every *let-7* gene cluster. These fragments are mostly associated with H3K acetylation (H3K27Ac), which marks transcriptional active regions within the genome (Supplementary Table 2). Moreover, LIP reduces transcription while LAP increases the transcription from a 2,6 kb C/EBPβ/H3K27Ac associated promoter-region of the *let-7a-1/f-1/d* cluster using a luciferase reporter assay previously described by others^37^ (Fig. 5d, e). These data indicate that the C/EBPβ isoforms transcriptionally regulate *let-7* microRNA clusters.

To investigate whether transcriptional repression of *let-7* by LIP precedes activation of the *let-7* target *Lin28b* we generated MEFs with cumate-inducible LIP expression. Following LIP induction, the levels of *let-7d*, *g* and *i* start to decrease 16 h after LIP-induction (Fig. 5f and Supplementary Fig. 4d), while *Lin28b-*mRNA levels were found increased only 48 h after LIP induction (Fig. 5g), supporting a mechanism where transcriptional repression of *let-7* by LIP precedes the activation of *Lin28b*.

Next, we asked how the LIP-*let-7* regulation correlates with LIP-induced changes in the proteome. Sixty eight of the 551 proteins found upregulated in LIP overexpressing cells have predicted *let-7* binding sites in their mRNA as was retrieved from the mirna.org database (Good mirSVR score^38^) (Fig. 5h). An analysis of their gene ontology did not show any specific metabolic pathway or metabolic cellular process (Supplementary Fig. 4e), suggesting that Lin28b is the main *let-7* target and mediator of metabolic regulation.

### LIP regulates the *let-7/Lin28b* circuitry in vivo

To evaluate regulation of *let-7* and *Lin28b* by C/EBPβ-LIP in vivo we generated a conditional LIP overexpression mouse model. A LIP expression cassette preceded by a floxed transcriptional stop cassette was integrated in the Rosa26 locus (Supplementary Fig. 5a). Intercrossing with the general Cre-deleter mouse line pCX-Cre^39^ (Supplementary Fig. 5b) resulted in mice with LIP overexpression in the investigated tissues bone marrow, skin and spleen of the further referred to as *R26LIP* mice (Supplementary Fig. 5c).

**Fig. 6:**
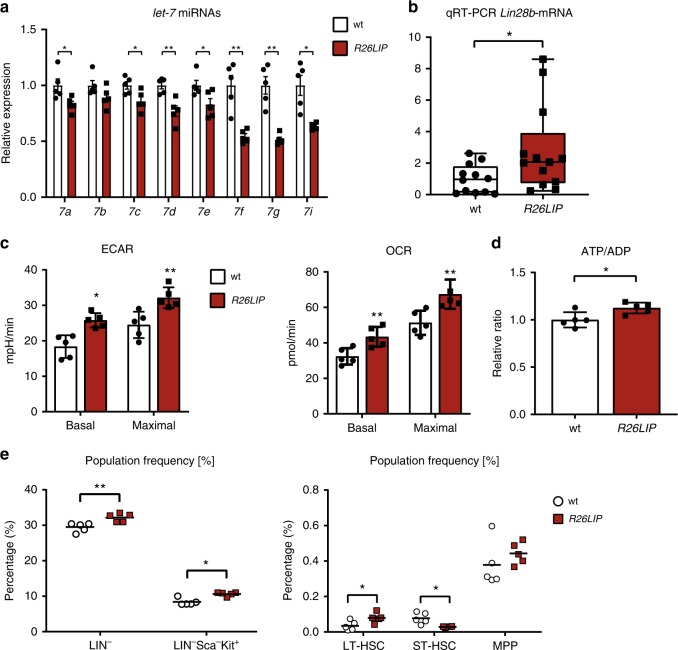
LIP regulates *let-7/Lin28b* in the bone marrow. **a** Expression levels of *let-7a, b, c, d, e, f, g* and *i* in bone marrow of *R26LIP* mice compared to wt mice (*n* = 5). **b** Expression level of *Lin28b*-mRNA by qRT-PCR in bone marrow of *R26LIP* mice compared to wt mice (*n* = 13). **c** ECAR and OCR of bone marrow cells of *R26LIP* mice compared to wt mice (*n* = 5). **d** Relative ATP/ADP ratio in bone marrow cells of *R26LIP* mice compared to wt mice (*n* = 5). **e** Population percentages of LIN^−^ and LIN^−^Sca^+^Kit^+^ cells, or LT-HSCs, ST-HSCs and MPPs in the bone marrow of *R26LIP* LIP-overexpressing mice compared to wt control mice (*n* = 5). Statistical differences were analysed by Student’s *t*-tests. Error bars represent +SD, *P < 0.05, **P < 0.01

To investigate in vivo effects of LIP upregulation we isolated bone marrow of *R26LIP* mice since these are suitable for Seahorse XF96 metabolic flux analysis. In the *R26LIP* bone marrow cells *let-7* miRs (*a, c, d, e, f, g, i*) were repressed and the Lin28b-mRNA was upregulated in comparison with bone marrow cells derived from wt control mice (Fig. 6a, b). Extracellular metabolic flux analysis (Seahorse XF96) revealed that *R26LIP* BM cells have increased OCR, ECAR (Fig. 6c) and ATP/ADP ratios (Fig. 6d). These in vivo results are reminiscent to the LIP-mediated metabolic alterations found in the studied cell lines (Fig. 1 and Supplementary Fig. 1). To examine whether the increased LIP expression would result in differences in bone marrow composition we performed flow cytometry analysis after staining bone marrow cells with antibodies specific for lineage and differentiation-stage specific surface markers. The percentage of lineage negative cells (LIN^−^) representing undifferentiated haematopoietic stem- and multipotent progenitor cells was increased in the bone marrow from LIP overexpressing mice compared to control mice, suggesting that the composition of the haematopoietic compartment in the bone marrow of LIP overexpressing mice is more immature (Fig. 6e and Supplementary Fig. 6a, b). In addition, we observed an increase in the percentage of lineage-negative, Sca1-negative, c-kit-positive myeloid progenitors (LIN^−^Sca^−^Kit^+^) in LIP overexpressing mice. Further analysis of the LIN^−^Sca^+^Kit^+^ fraction of bone marrow cells revealed that the percentage of long-term hematopoietic stem cells (LT-HSCs, CD48^−^ CD150^+^) which have life-long self-renewal potential and are believed to be the most immature cells in the bone marrow, were increased in the bone marrow from LIP overexpressing mice (Fig. 6e and Supplementary Fig. 6a, b). In contrast, the fraction of short-term HSCs (ST-HSCs, CD48^−^ CD150^−^) that only have limited self-renewal potential was reduced. The percentage of multipotent progenitors (MPPs, CD48^+^ CD150^−^) that lack self-renewal capacity is not significantly changed in the LIP overexpressing mice. Although these analyses do not completely resolve the alterations in the bone marrow that are induced by LIP overexpression, they point towards a higher percentage of immature cells in the LIP overexpressing mice.

**Fig. 7:**
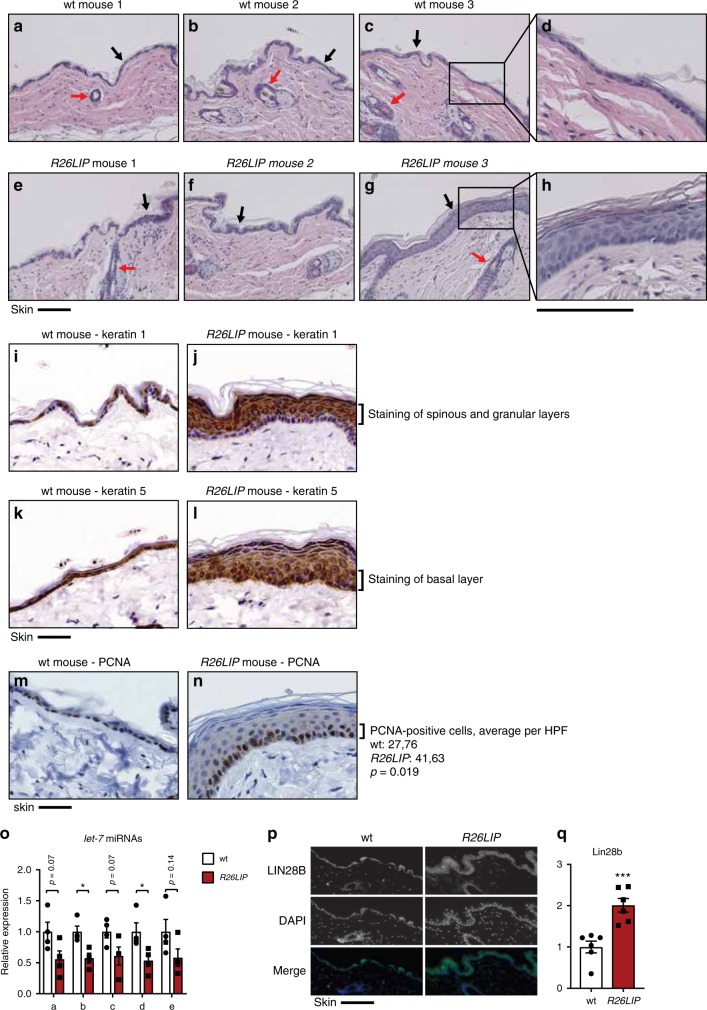
LIP regulates *let-7/Lin28b* in the epidermis. **a**–**d** Bright-field microscopy images of haematoxylin and eosin (HE) stained skin sections of wt (**a**–**d**) and *R26LIP* (**e**–**h**) mice. Black arrows point to epidermis; red arrows to hair follicles. Scale bars represent 100 μm. Bright-field microscopy images of Keratin 1 immunohistochemical staining of skin sections of wt (**i**) and *R26LIP* (**j**) mice, and of Keratin 5 staining of skin sections of wt (**k**) and *R26LIP* (**l**) mice. Scale bars represent 50 μm. Bright-field microscopy images of PCNA immunohistochemical staining of skin sections of wt (**m**) and *R26LIP* (**n**) mice, with quantification at the right (*n* = 5 for wt and *n* = 6 for R26LIP, 5 HPF per mouse). **o** Expression levels of let-7a, b, c, d and e in the skin of *R26LIP* mice compared to wt mice (*n* = 4). **p** LIN28B specific immunofluorescence microscopy images of skin sections of a *R26LIP* mice and wt mice. Scale bar 100 μm. **q** The bar graph shows quantification of LIN28B-staining (*n* = 6). Statistical differences were analysed by Student’s *t*-tests. Error bars represent SD, *P < 0.05, **P < 0.01, ***P < 0. 001

C/EBPβ, its paralogue C/EBPα and LIN28 have been shown to be key regulators in skin differentiation and hair follicle function in separate studies. C/EBPβ and C/EBPα are expressed in the nuclei of basal keratinocytes and their expression is upregulated during differentiation with the highest expression in the stratum granulosum. Double knockout of the functionally redundant *Cebpa* and *Cebpb* genes in skin revealed that they are essential for, the coupling between cell cycle exit and commitment of epidermal differentiation through inhibition of E2F, the restriction of stem cell function through downregulation of stem cell signature genes, and the expression of key molecules in epithelial barrier function^40–42^. Transgenic expression of *Lin28a* in mice results in the development of a thicker skin and thicker hair coats due to an expanded anagen phase (active growth phase) in the hair follicles^6,43^. We observed a similar epidermal thickening (in five of six examined animals) and a shift towards anagenic hair follicles (in four of six examined animals) in *R26LIP* mice that overexpress LIP (Fig. 7a–h and Supplementary Table 3). Staining for Keratin 1 and 5 showed that the stratification of the thickened epithelial tissue in the *R26LIP* mice is preserved; Keratin 1 is expressed in the spinous and granular layers and the strongest staining for keratin 5 is found in the basal layer (Fig. 7i–l). Staining for proliferating cell nuclear antigen (PCNA) showed an increase in proliferating cells and a higher cell density in the basal layer (average PCNA-positive cells per high-power field (HPF); 41.63 for *R26LIP* mice and 27.76 for wt, *P* = 0.0187) (Fig. 7m, n). Taken together, these data suggest that higher proliferation of keratinocytes in the basal layer causes the thickening of the spinous layer in *R26LIP* mice.

In accordance with our in vitro results and results from the bone marrow of *R26LIP* mice expression of the *let-7* miRs (*a, b, c, d, e*) was reduced in isolated skin of *R26LIP* mice (Fig. 7o) and immunohistochemical staining showed an increase in LIN28B protein levels in the epidermis of *R26LIP* mice compared to control mice (Fig. 7p, q). Thus, our data show that upregulation of LIP results in suppression of *let-7* and consequently activation of *Lin28b* in the skin, which results in epidermal thickening and an increase in anagen hair follicles. Taken together we show that LIP regulates the *let7*/LIN28B circuitry in vivo, which is associated with metabolic reprogramming toward enhanced glycolysis and mitochondrial respiration and results in tissue hyperplasia.

## Discussion

Here we show that the transcriptional activator C/EBPβ-LAP and the transcriptional inhibitor C/EBPβ-LIP differentially regulate cellular metabolism. LIP robustly enhances both the extracellular acidification rate (ECAR) as a measurement of aerobic glycolytic flux and the oxygen consumption rate (OCR) as a measurement for mitochondrial respiration, while LAP only enhances respiration (OCR) but to a lesser extent than observed with LIP. Expression of LIP also increases the mitochondrial mass, which likely contributes to the increase in respiration capacity in cells with high LIP expression. The metabolic alterations induced by LIP support cell growth and proliferation and are characteristic for cancer cells^2^. Mechanistically, we showed that LIP requires the RNA-binding protein LIN28B for its metabolic reprogramming function and that LIP upregulates LIN28B through transcriptional downregulation of *let-7* microRNAs that are known to target the lin28b-mRNA. By using the LIP-overexpressing transgenic *R26LIP* mouse model we could recapitulate the LIP-*let7*-LIN28B regulation in vivo which resulted in similar metabolic changes in *R26LIP* bone marrow cells as was found in cell culture experiments. Furthermore, the occurrence of hyperplasia in the skin of the *R26LIP* mice suggests that aberrant induction of the LIP-*let-7*-LIN28B pathway provokes the deregulation of tissue homeostasis and therefore could support a pro-tumourigenic state.

Earlier experiments using different mouse models have revealed a key role of C/EBPβ in the regulation of organismal metabolism; for example C/EBPβ deficient mice are protected against high fat diet induced obesity^44^. We previously showed that mice deficient in LIP expression but normal in LAP expression consume more oxygen and display a metabolic shift away from carbohydrate use toward more fatty acid oxidation, which is accompanied by metabolic improvements reminiscent with those found under calorie restriction^24^. Here, we show that at the cellular level LAP enhances respiration but not glycolysis. LIP on the contrary robustly increases both rate and capacity of aerobic glycolysis and mitochondrial respiration—a condition that resembles cancer cell metabolism. Omics analysis revealed that LIP does not significantly alter relevant mRNA levels in glycolytic pathways, but instead the levels of enzymes of the glycolytic and pentose phosphate pathways are elevated. Our study points to an important role of the LIP-*let-7*-LIN28B controlled metabolic regulation in the context of proposed oncogenic functions of LIP in vitro and in vivo^22,25,27^. The regulation of LIN28B by LIP-*let-7* predicts that the paralogue LIN28A is similarly regulated because both Lin28a- and Lin28b-mRNAs are known *let-7* targets. While in the main experimental system used in this study (MEFs) LIN28A is not expressed and could not be studied, we do observe strong upregulation of LIN28A by LIP in T47D cells (Supplementary Fig. 4a), suggesting that indeed LIN28A can be part of the LIP-*let-7*-LIN28A/B regulatory circuit.

Eight conserved clusters in the mouse or human genomes encode for the family of nine functionally redundant *let-7* microRNAs (*a, b, c, d, e, f, g, i* and *miR-98*)^18,45^. This redundancy is one of the reasons for the strong tumour suppressive functions of *let-7*. The transcriptional regulation of *let-7* in vertebrates is not well understood. It is assumed that factors which regulate transcription of several if not all clusters would be most effective in regulating the collective function of the *let-7* microRNAs^18^. In this study we reveal that LIP is such a master regulator of *let-7* levels and thereby activates LIN28B.

**Fig. 8:**
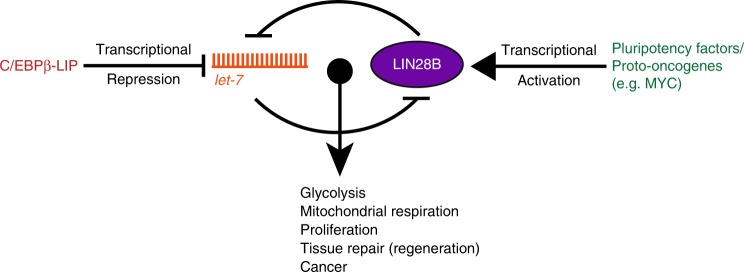
Model of a dual mode of regulating the *let-7/*LIN28B circuit. The transcriptional activation of LIN28B by pluripotency factors and proto-oncogenes and the transcriptional repression of *let-7* by C/EBPβ-LIP dually control the influence of the LIN28B/*let-7* circuit on cellular metabolism, tissue repair and cancer

Apart from the role of the *let-7* miRs relatively little is known about the (post-)transcriptional regulation of Lin28a/b-mRNAs. It is reported, that Lin28a/b-mRNAs are suppressed by microRNA *mir-125*/*lin-4* during stem cell differentiation^46^, that pluripotent factors transcriptionally upregulate Lin28a in mammalian ESCs^47^, and that Myc or NF-kB can transcriptionally activate Lin28b in cancer cells^45,48,49^. Our experiments did not reveal direct transcriptional regulation by LIP of the *Lin28b*-promoter, nor does the ENCODE database record high-score C/EBPβ-associated DNA fragments (http://genome.ucsc.edu/ENCODE/) at the *Lin28b*-promoter. Since, repression of *let-7* and/or activation of *Lin28a/b* will modulate the *let-7*/LIN28A/B circuit, regulatory factors like MYC and LIP would act either alone, or together while reinforcing each other’s function, contribute to low *let-7*/high Lin28A/B-driven metabolic reprogramming, proliferation and tumourigenesis^48^ (see model Fig. 8).

We have shown before that expression of LIP, involving a *cis*-regulatory small uORF in the Cebpb-mRNA, is suppressed by treatment with the mTORC1-inhibitor rapamycin^24^. In addition, we have identified other FDA approved drugs in a screening approach that suppress LIP^50^. Therapeutic interventions that suppress LIP and thereby impair cancer cell metabolism could complement existing cancer therapies. Furthermore, interfering with glycolysis or mitochondrial respiration much stronger affected proliferation of LIP expressing cells compared to LAP expression or empty vector control cells (Fig. 2d). Thus, high LIP expression could be a feature exploited for cancer diagnosis, revealing those cancer types that are specifically sensitive to treatment with metabolic inhibitors that are already in pre-clinical or clinical development^51^. In contrast to LIP expressing cells, LAP expressing cells seem to be resistant against rotenone treatment (Fig. 2e). This might be explained by our earlier observation that LAP induces fatty acid oxidation^24^. While the NADH generated during fatty acid oxidation enters the respiratory chain at complex 1, which is inhibited by rotenone, the generated FADH2 enters at complex II whose function is unaffected by rotenone and thus could maintain sufficient energy levels for survival and/or proliferation of these cells.

Our transcriptome analysis revealed that LIP downregulates gene clusters with functions in the extracellular matrix, cell adhesion and polysaccharide binding. This suggests an oncogenic function of LIP in cancer cell migration, which is in line with a study showing that breast cancer cells with high LIP levels are more aggressive and prone to migrate^29,52^. Metabolic reprogramming has been linked to metastatic potential in breast cancer^53^. Because C/EBPβ-LIP is involved in the regulation of cell migration and cellular metabolism it could be a determining factor for the metastatic capacity by co-regulating cell migration and metabolism.

In the *R26LIP* mouse model we did not observe an increased incidence of cancer at the investigated age of 9–12 months. However, elevated LIP expression in a different knockin mouse model was associated with an increased cancer incidence upon ageing^33^. Therefore, LIP overexpression may not be sufficient to initiate cancer development but collaborate with additional oncogenic mutations and/or with age-related pathophysiological changes to induce or support tumourigenesis. Since *Lin28b* upregulation is critically involved in the development of different tumour types it likely contributes to tumour development and/or maintenance in tumours in which LIP levels are increased.

C/EBPβ is a known regulator of epidermal differentiation^40–42^ and of the anagen growth phase in hair follicles^40,54^. However, the role of the different C/EBPβ protein isoforms was not addressed in those studies. The development of skin hyperplasia in the *R26LIP* mice suggests that LIP counteracts the differentiation functions of LAP and probably also interferes with the function of C/EBPα. The LIP overexpression in *R26LIP* mice is correlated with *let-7* repression and LIN28B upregulation in the skin. Since a similar skin hyperplasia phenotype is found in LIN28A overexpressing mice^6^ the *let-7*-LIN28 regulation is likely involved in the epidermal functions of C/EBPβ. C/EBPβ is expressed in hematopoietic cells of different lineages and differentiation stages. Overexpression of LIP in the *R26LIP* mice with the concomitant increase in ECAR (glycolysis) and OCR (respiration) in the bone marrow was expected to affect haematopoiesis. Overall, the data point towards a moderate increase of immature cells in the LIP overexpressing mice although the reduction in short-term haematopoietic stem cells (ST-HSC) could also reflect a more efficient differentiation into lineage specific progenitor cells. For more complete functional analysis of the LIP-induced alterations in the hematopoietic system more elaborate experiments must be performed.

C/EBPβ and *let-7* are expressed in a wide range of adult tissues (www.genecard.org) and animals with transgenic expression of *let-7* or reduced levels of LIP have reduced liver regeneration capacities after partial hepatectomy^23,55^. In 3T3-L1 cells, a cellular model for adipocyte differentiation it was shown that *let-7* expression increases upon terminal differentiation into adipocytes^56^. In the same model, overexpression of C/EBPβ-LIP results in disturbed differentiation and cellular transformation^22^. Our work provides a link between these observations showing that LIP represses *let-7* expression while the transcriptionally potent LAP (and eventually other C/EBP family members) up-regulate *let-7* expression^57^, thereby regulating the transition between proliferation and terminal differentiation in different tissues. Furthermore, our findings raise the question if C/EBPβ-*let-7*-LIN28B regulation plays a general role in tissue maintenance and regeneration.

Taken together, we identified LIP as regulator of the *let-7*/LIN28B circuit, which induces a metabolic shift that is a characteristic of cancer cells. The possibility to target the translational expression of LIP^50^ may open up opportunities to interfere with the *let-7*/LIN28B circuit in tissue homeostasis and cancer.

## Methods

### Cell culture

HEK293T cells, Hepa1-6 cells, BT20 cells and all immortalized MEF cell lines were cultured in high glucose DMEM supplemented with 10% FBS, 10 mM HEPES, 1 mM Sodium Pyruvate and 100 U/ml Penicillin Streptomycin. T47D and MCF7 breast cancer cells were maintained in RPMI1640 medium supplemented with 10% FBS, 25 mM HEPES, 1 mM Sodium Pyruvate and 100 U/ml Penicillin/Streptomycin. C/EBPβ^ΔuORF^ MEF, *Cebpb* ko MEF and *p53* ko MEF were described before^24^.

### DNA constructs

Plasmids containing rat C/EBPβ-LAP, rat C/EBPβ-LIP and flag-tagged rat C/EBPβ-LIP were described before^24^. For overexpression of human C/EBPβ-LIP, the coding sequence was amplified from MCF7 genomic DNA (forward primer: 5’- CCGAGCTCAAGGCGGAGCC-3′, reverse primer: 5′- TAAAATTACCGACGGGCTCCCC-3′). The amplified PCR product was cloned into pcDNA3.1 (Invitrogen) and checked for mutation by Sanger sequencing.

### Transfection

Immortalized MEFs were transfected with an empty, rat C/EBPβ-LIP or -LAP containing pcDNA3 or pSV2Zeo vector by using Fugene HD (Promega) according to the manufactures protocol. For stable overexpression, C/EBPβ-ko MEFs were treated with 0.2 mg/ml Zeocin (Invitrogen). To maintain the expression cells were culture with 0.1 mg/ml Zeocin in the medium. Immortalized wt MEFs transfected with empty pcDNA3 or LIP-containing pcDNA3 were selected with 0.8 mg/ml G418 and to maintain the overexpression cultured with 0.5 mg/ml G418. BT20 cells, T47D cells and MCF7 cells were transfected with empty or human LIP-containing pcDNA3.1 via Fugene HD (Promega) using the manufactures protocol. For stable expression, MCF7 cells were selected with 0.8 mg/ml, T47D with 0.4 mg/ml and BT20 with 1.2 mg/ml G418. For lentivirus production, 4.5 × 10^6^ HEK293T cells were plated in 10-cm culture dishes. Twenty-four hours later, transfection was performed using the calcium phosphate method.

### Lentiviral transduction

P53^−/−^ MEFs were infected with a cumate-inducible C/EBPβ-LIP-FLAG construct or an empty vector construct using a standard protocol. Two days after infections cells were selected with puromycin (1.66 µg/ml). Hepa1-6 cells were infected with an empty or C/EBPβ-LIP-FLAG containing pLVX-IRES-neo vector and selected with 0.9 mg/ml G418.

### CRISPR/Cas gene targeting

A gRNA against mouse *Lin28b* was designed (5′-CATCTCCATGATAAGTCGAGAGG-3′) and cloned into pSpCas9(BB)-2A-GFP (PX458, Addgene plasmid #48138). Two days after electroporation (Lonza, Amaxa MEF2 Nucleofector Kit, protocol T-20) GFP-positive cells were sorted and plated at low density (65 cells per mm^2^) to form single cell colonies. After 2 weeks of culture colonies were transferred to single wells of a 96-well plate and expanded. Several clones were isolated and the genomic *Lin28b* sequence was checked by Sanger sequencing. LIN28B protein expression was checked by immunoblot.

### Proliferation assays

5 × 10^4^ immortalized MEFs were seated in 6-cm dishes. After 3 days cells were trypsinised and counted using an automated cell counter (TC20, Biorad). Cell numbers were transformed to population doublings (Formula: ${\mathrm{Population}}\,\mathrm{doubling} = \frac{{\mathrm{log}10\left( {\frac{{\mathrm{final}}\,{\mathrm{cell}}\,{\mathrm{number}}}{{\mathrm{starting}}\,{\mathrm{cell}}\,{\mathrm{number}}}} \right)}}{{\mathrm{log}10\left( 2 \right)}}$)

For 2-Deoxyglucose (2-DG) and Rotenone treatment *Cebpb*-ko MEFs with control empty vector (EV) or ectopic expression of LIP or LAP were seeded in 96-well plates (2000 cells per well) in the presence of different concentrations of Rotenone (0 nM; 100 nM; 250 nM; 500 nM) or 2-DG (0 mM; 1 mM; 2.5 mM; 5 mM; 10 mM) and relative viable cell numbers were determined three days later using the CellTiter-Fluor^TM^ cell viability assay (Promega) according to the instructions of the manufacturer.

### Metabolic flux analysis

Metabolic flux analysis was performed using a Seahorse XF96 Extracellular Flux analyser (Agilent Bioscience). 1.5 × 10^4^ immortalized MEFs were seeded 4 h before the assay. Cancer cell lines were seeded with different densities (Hepa1-6, T47D: 3 × 10^4^; BT20: 1.5 × 10^4^) 16 h before the experiment. For the Seahorse flux analysis of bone marrow cells, Seahorse plates were treated with poly-L-lysine. 4 × 10^5^ freshly isolated bone marrow cells were centrifuged into the poly-l-lysine layers of a Seahorse plate just before the start of the assay. Assays were performed according to the manufactures protocol. Injected drugs were oligomycin (2.5 µM) for maximal glycolytic capacity/inhibition of ATP dependent mitochondrial oxygen consumption, 2,4-Dinitrophenol (50 µM) for maximal oxygen consumption and 2-deoxyglucose (100 mM) for inhibition of glycolysis.

### Mitochondrial analysis

Mitochondrial mass was determined by staining the cells with the MitoTracker Red 480 kit (Thermo Fisher Scientific) according to the instructions of the manufacturer, fixed with paraformaldehyde (3.7%), permeabilized by incubation in ice-cold acetone, counterstained with DAPI as a control for actual cell numbers and mounted on glass slides with Pro-Long Gold Antifade Reagent (Thermo Fisher Scientific). Photographs of the fluorescent cells from different microscopic fields were taken using a Leica DMI 6000 B fluorescence microscope with the LAS AF software and quantified as ratio between Mitotracker 480- and DAPI staining using the ImageJ software.

Mitochondrial and genomic DNA were co-purified using a standard protocol and the ratio between mitochondrial and genomic DNA was determined by qPCR using the LightCycler 480 SYBR Green I Master Mix (Roche) and a cytochrome b gene specific primer pair (for: CAT TTA TTA TCG CGG CCC; rev: TGT TGG GTT GTT TGA TCC TG) for mitochondrial DNA and a β-actin gene specific primer pair (for: AGA GGG AAA TCG TGC GTG AC; rev: CAA TAG TGA TGA CCT GGC CGT) for genomic DNA.

### Luciferase-based assays

NADH, NAD+, ATP and ADP levels were distinguished using luciferase-based assays. Twenty-four hours before the assay, 5000 cells per well were seeded in a 96-well plate. Experiments were performed according to manufactures protocols (NADH/NAD+: Promega, G9071; ATP/ADP: Biovision, K255-200).

For the luciferase-based promoter assay 2500 p53^−/−^ MEFs were seeded 3 days before the measurement. Two days prior the measurement cells were transfected with empty, human LAP or human LIP containing pcDNA3.1, empty or *let-7a-1/f-1/d* promoter containing pGL4.23^37^ and pGL4.73-renilla (for normalization) using Fugene HD (Promega). Just before the measurement the medium was removed and phenol red free DMEM was added. The measurement of firefly and renilla activity was performed according to the manufactures protocol (Promega, E1910). For detection, a GloMax-Multi Detection System (Promega) was used.

### qRT-PCR analysis

Total RNA was isolated using the miRNeasy Isolation Kit (Qiagen) following the manufactures protocol. For the analysis of microRNA expression, cDNA was synthesized from 500 ng RNA using the miScript II RT kit (Qiagen). qRT-PCR was performed using the miScript SYBR green PCR kit (Qiagen) and commercially available primers for *let-7a, b, c, d, e, f, g* and *i* (Qiagen). Primers for Snord72 and U6 were used for normalization. For the analysis of mRNA expression, 500 ng RNA were reverse transcribed with random hexamer primers using the transcriptor first strand cDNA synthesis kit (Roche). qRT-PCR was preformed using the LightCycler 480 SYBR Green I Master Mix (Roche) and commercially available mouse and human *Lin28b* primers (Qiagen).

### Analysis of differentially expressed genes

In general, sequencing was done using the next-generation sequencing technology of Illumina^58^. RNA was isolated from stable overexpressing C/EBPβ-LIP, C/EBPβ-LAP and empty vector control C/EBPβ ko fibroblasts using the RNeasy Plus Mini Kit (QIAGEN). Quality and quantity of RNA was determined using Agilent’s Bioanalyzer 2100 in combination with the RNA 6000 nano chip (Agilent). Library preparation was done using Illumina’s TruSeq RNA v2 following the manufacturer’s description. The libraries were quality checked and quantified using Bioanalyzer 2100 (as mentioned above) in combination with the DNA 7500 kit (Agilent). Sequencing was done on a HiSeq2500 in 50 bp, single-end sequencing, high-output mode. Sequence information was extracted using bcl2fastq v1.8.3 (Illumina), Sequencing resulted in around 50 mio reads per sample. Obtained reads were aligned to the GRCh38/mm10 genome (refSeq annotation from 2012-05-17) using TopHat v1.4.1^59^ and quantified using the HTSeq count v0.5.4^60^. Identification of differentially expressed genes (DEGs) was done by using the bioconductor packages (https://www.bioconductor.org/) edgeR^61^ and DESeq^62^ in the statistical environment R. The resulting *p*-values were adjusted using Benjaminin and Hochberg’s approach for controlling the false discovery rate^63^. Genes were regarded as differentially expressed if adjusted *p*-values of edgeR and DEseq < 0.05. The data were analysed using R/bioconductor or Perseus software (http://www.coxdocs.org/). Functional clustering analysis was performed using the DAVID database (https://david.ncifcrf.gov/, version 6.7) at default settings with high stringency.

### Proteome analysis

*Cebpb*-ko fibroblasts stably overexpressing C/EBPβ-LIP, C/EBPβ-LAP or empty vector control were lysed using a Urea-Lysis-Buffer (8M Urea, 100 mM Tris pH 8.4). 100 μg of proteins were reduced in DTT 2 mM for 30 min at 25 °C and successively free cysteines were alkylated in 11 mM iodoacetamide for 20 min at room temperature in the darkness. LysC digestion was performed by adding LysC (Wako) in a ratio 1:40 (w/w) to the sample and incubating it for 18 h under gentle shaking at 30 °C. After LysC digestion, the samples were diluted three times with 50 mM ammonium bicarbonate solution, 7 µl of immobilized trypsin (Applied Biosystems) were added and samples were incubated 4 h under rotation at 30 °C. Digestion was stopped by acidification with 5 μl of trifluoroacetic acid and trypsin beads were removed by centrifugation. Fifteen micrograms of digest were desalted on STAGE Tips, dried and reconstituted to 20 µl of 0.5% acetic acid in water^64,65^. We used a LC-MS/MS system (NanoLC 400 [Eksigent] coupled to Q Exactive HF [Thermo]) and a 240 min gradient ranging from 5 to 40% of solvent B (80% acetonitrile, 0.1% formic acid; solvent A = 5% acetonitrile, 0.1% formic acid) to analyse 5 μl of each sample in duplicate. A MonoCap C18 HighResolution 2000 (GL Sciences) of 100 cm length was used for the chromatographic separation. The nanospray source was operated with an ion transfer tube temperature of 260 °C and a spay voltage of 2.4 kV. The data dependent mode was used to acquire the data with a top 10 method (one survey MS scan with resolution 70,000 at *m/z* 200, followed by up to 10 MS/MS scans on the most intense ions with intensity threshold 5000). In order to increase new sequencing events ions, once selected for fragmentation ions were excluded from further selection for 45 s. Raw data were analysed using the MaxQuant proteomics pipeline (v1.5.3.30) and the built in the Andromeda search engine^66^ with the mouse Uniprot database. Carbamidomethylation of cysteines was chosen as fixed modification, oxidation of methionine and acetylation of N-terminus were chosen as variable modifications. The search engine peptide assignments were filtered at 1% FDR and the feature match between runs was enabled; other parameters were left as default. For statistical analysis and visualization of the data, Perseus software was used at default settings. Functional clustering analysis was performed using the DAVID database (https://david.ncifcrf.gov/, version 6.7) at default settings with high stringency.

### Immunoblot analysis

Cells and tissues were lysed using RIPA buffer. Equal amounts of protein were separated via SDS-PAGE and transferred to a PVDF membrane using Trans-Blot Turbo System (Bio-rad). The following antibodies were used for detection: C/EBPβ (E299) from Abcam; LIN28B (mouse preferred) from Cell Signaling Technology, β-tubulin (GT114) from GeneTex and β-actin (clone C4) (#691001) from MP Biomedicals. For detection, HRP-conjugated secondary antibodies (Amersham Life Technologies) were used. The signals were visualized by chemiluminescence (ECL, Amersham Life Technologies) using ImageQuant LAS 4000 mini imaging machine (GE Healthcare Bioscience AB) and the supplied software was used for the quantification of the bands.

### Generation of the transgenic mouse line R26LIP

The coding sequence for C/EBPβ-LIP was cloned into TV-Rosa26-LMP1/CD40^67^ via AscI restriction sites. 129/SV ES cells were transfected with TV-Rosa26-C/EBPβ-LIP and selected with G418 for 7 days. Clones were tested for integration of the target vector by PCR (fw1: 5′-AGG ACA ACG CCC ACA CAC CAG GTT AGC-3′, fw2: 5′-AGT TCA TCA CGC GCT CCC ACT TGA AGC C-3′,rv: 5′-TTT GGG GCT CCG GCT CCT CAG AGA GC-3′) and southern blot (digest of gDNA with MfeI and detection of specific DNA fragment with radioactive probe). One positive clone was injected into C57BL/6 blastocysts and chimeras were backcrossed with C57BL/6 to start the *R26-iCBLIP* mouse line. *R26-iCBLIP* mice were crossed with general Cre-deleter mouse line pCX-Cre^39^ to generate the *R26LIP* mouse line that was used for experiments.

### Animals

Experimental male mice were housed at a standard 12-h light/dark cycle at 22 °C in a pathogen-free animal facility for 9–12 months. Numbers of mice used in the separate experiments are indicated in the figure legends. *R26-LIP* mice were backcrossed for four generations into the C57BL/6J genetic background. All of the animals were handled according to approved institutional animal care and use committee (IACUC) protocols of the University of Groningen (#6996A).

### Bone marrow isolation and FACS analysis

Bones of the legs, spin and sternum were cleaned from soft tissues and carefully homogenised in red blood cell lysis buffer (155 mM NH_4_Cl, 12 mM NaHCO_3_ and 0.1 mM EDTA) by pestle and mortar. After addition of one volume of 0.2% bovine serum albumin (BSA) in PBS, cell suspensions were spin down at 1400 RPM for 5 min at 4 °C. Cell pellets were resuspended in 1 ml of 0.2% BSA in PBS and cell numbers were determined using TC20 cell counter (Bio-Rad). Equal numbers of bone marrow cells were incubated with an antibody mixture (Sca1-Pacfic blue, c-Kit-phycoerythrin, CD150-phycoerythrin/Cyanine7, CD48-Alexa 647 and lineage markers-Alexa 700: Ter119, CD11b, CD3e, B220, and Gr1, all from Bio Legend) for 40 min at 4 °C in the dark, washed and resuspended using 0.2% BSA in PBS. Cell populations were analysed with a BD FACSCanto II Flow Cytometer (BD Bioscience) using the gating strategy shown in Fig. S6.

### Histology

Pieces of tissue were fixed with 3.7% paraformaldehyde for 48 h and embedded in paraffin. Five micrometres-thick sections were stained with haematoxylin and eosin (H&E) and analysed by a pathologist. For immunofluorescent and immunohistochemical staining, 5 µm-thick (in the case of PCNA staining 4 µm-thick) sections were backed over night at 55 °C, deparaffinized and rehydrated. For antigen retrieval, skin sections were incubated overnight (15 min in case of PCNA staining) in 10 mM citrate buffer at 60 °C. For the detection of Keratin 1 and Keratin 5, slides were stained with specific antibodies (anti-Keratin 1: Biolegend, #905601, 1:1000 and anti-Keratin 5: Biolegend, #905501, 1:4000) in the DAKO Autostainer (Dako) using a standard protocol. For the detection of PCNA, endogenous peroxidase activity was blocked with 1% H_2_O_2_ in methanol and slides were stained using the VECSTAIN ABC kit (Vector # PK4000, 1:25), the anti PCNA antibody (Abcam # AB92552, 1:600) and a biotin-coupled goat anti rabbit secondary antibody (Vector # BA-1000, 1:250). Slides were then counterstained with haematoxylin, dehydrated and covered using Eukitt. The anti-LIN28B staining was performed using the Tyramide SuperBoost™ Kit (Thermo Fisher Scientific, # B40941) and the anti-LIN28B antibody was used in a concentration of 1:100 (Cell Signaling Technology, # 5422S). Skin sections were stained with DAPI and mounted with non-hardening mounting medium. Images were taken using a TissueFAXS microscope (TissueGnostics). Fluorescence signals were analysed via the supplied software and ImageJ (https://imagej.nih.gov/ij/).

### Statistics and reproducibility

For analysis of differentially expressed genes (transcriptome) and proteome we refer to the specific sections above. Other statistical differences were calculated by Student’s *t*-test using GraphPad Prism 8. Data are presented as the mean ± standard deviation (SD). Statistically significant differences are indicated with *p < 0.05, **p < 0.01, ***P < 0.001.

### Reporting summary

Further information on research design is available in the Nature Research Reporting Summary linked to this article.

## Supplementary information

Description of Supplementary Data

Supplementary Information

Reporting Summary

Supplementary Data 1

Supplementary Data 2

Supplementary Data 4

Supplementary Data 3

Supplementary Data 5

Supplementary Data 6

Supplementary Data 7

## Data Availability

RNA-sequencing data as presented in Fig. 2 and Supplementary Fig. 2 are available at NCBI’s gene expression omnibus (GEO, https://www.ncbi.nlm.nih.gov/geo/)^68^ with identifier GSE110316. The mass spectrometry proteomics data as presented in Fig. 2 have been deposited to the ProteomeXchange (http://www.proteomexchange.org) Consortium via the PRIDE^69,70^ partner repository with the dataset identifier PXD010095. The source data underlying main figures are presented in Supplementary Data 1–7.
