# Supplementary material for: C/EBPβ-LIP induces cancer-type metabolic reprogramming by regulating the let-7/LIN28B circuit in mice
Source: Commun Biol. 2019 Jun 14;2:208. doi: 10.1038/s42003-019-0461-z (PMC6572810; doi:10.1038/s42003-019-0461-z)
Supplement: Supplementary file 1 — Description of Supplementary Data [file 42003_2019_461_MOESM1_ESM.docx]

Description of the supplementary items:

The following excel files contain the source data underlying each figure.

Supplementary Data 1.xlsx (Fig 1).

Supplementary Data 2.xlsx (Fig 2).

Supplementary Data 3.xlsx (Fig 3).

Supplementary Data 4.xlsx (Fig 4).

Supplementary Data 5.xlsx (Fig 5).

Supplementary Data 6.xlsx (Fig 6).

Supplementary Data 7.xlsx (Fig 7).
