# Supplementary material for: C/EBPβ-LIP induces cancer-type metabolic reprogramming by regulating the let-7/LIN28B circuit in mice
Source: Commun Biol. 2019 Jun 14;2:208. doi: 10.1038/s42003-019-0461-z (PMC6572810; doi:10.1038/s42003-019-0461-z)
Supplement: Supplementary file 2 — Supplementary Information [file 42003_2019_461_MOESM2_ESM.pdf]

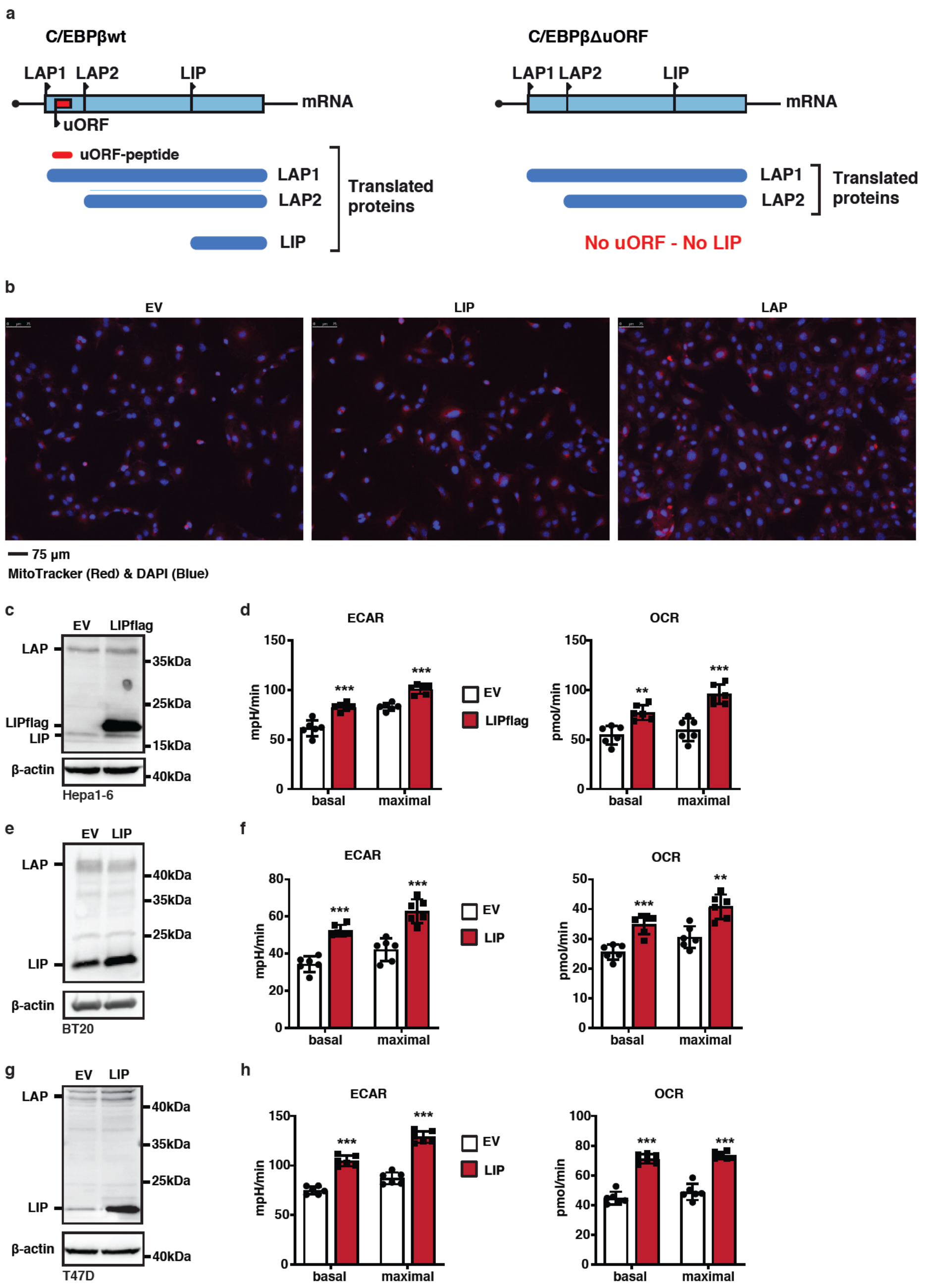

Supplementary Figure 1 C/EBPβ-LAP and -LIP isoforms regulate cellular metabolism. **a** Schematic representation of the C/EBPβ isoform expression in wt and C/EBPβ$^{\Delta uORF}$ MEFs. **b** Microscopic pictures from *Cebpb*-ko MEFs with control empty vector (EV) or ectopic expression of LIP or LAP stained wit MitoTracker (red) and DAPI (blue). **c** Immunoblot analysis of C/EBPβ-LAP and C/EBPβ-LIP expression in human hepatocellular carcinoma cell line Hepa1-6 ectopically expressing LIPflag compared to control (EV). β-actin is used for loading control. Uncropped images are shown in Supplementary Fig. 7. **d** ECAR and OCR of Hepa1-6 cells ectopically expressing LIPflag compared to control (EV)(n=6). **e** Immunoblot analysis of C/EBPβ-LAP and C/EBPβ-LIP expression in human breast cancer cell line BT20 ectopically expressing LIP compared to control (EV). β-actin is used for loading control. Uncropped images are shown in Supplementary Fig. 7. **f** ECAR and OCR of BT20 cells ectopically expressing LIP compared to control (EV)(n=6). **g** Immunoblot analysis of C/EBPβ-LAP and C/EBPβ-LIP expression in human breast cancer cell line T47D ectopically expressing LIP compared to control (EV). β-actin is used for loading control. Uncropped images are shown in Supplementary Fig. 7. **h** ECAR and OCR of T47D cells ectopically expressing LIP compared to control (EV)(n=6). Statistical differences were analyzed by Student's t-tests. Error bars represent ±SD, *P<0.05, **P<0.01, ***P<0. 001.

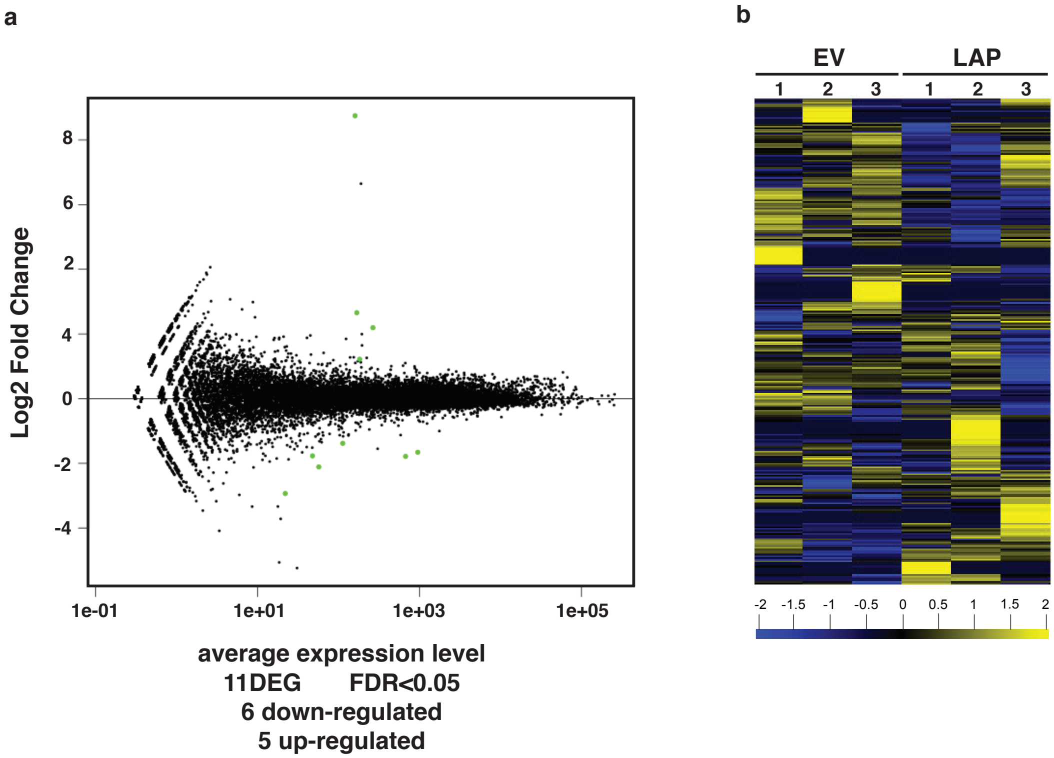

Supplementary Figure 2 LIP differential regulates transcriptome and proteome. **a** Differential expressed genes (DEG) by ectopic expression of LAP in *Cebpb*-ko MEFs compared to control (empty vector (EV)) with an FDR < 0.05. **b** Heatmap representation of differential expressed proteins by LAP expression in *Cebpb*-ko MEFs compared to EV control (z-score).

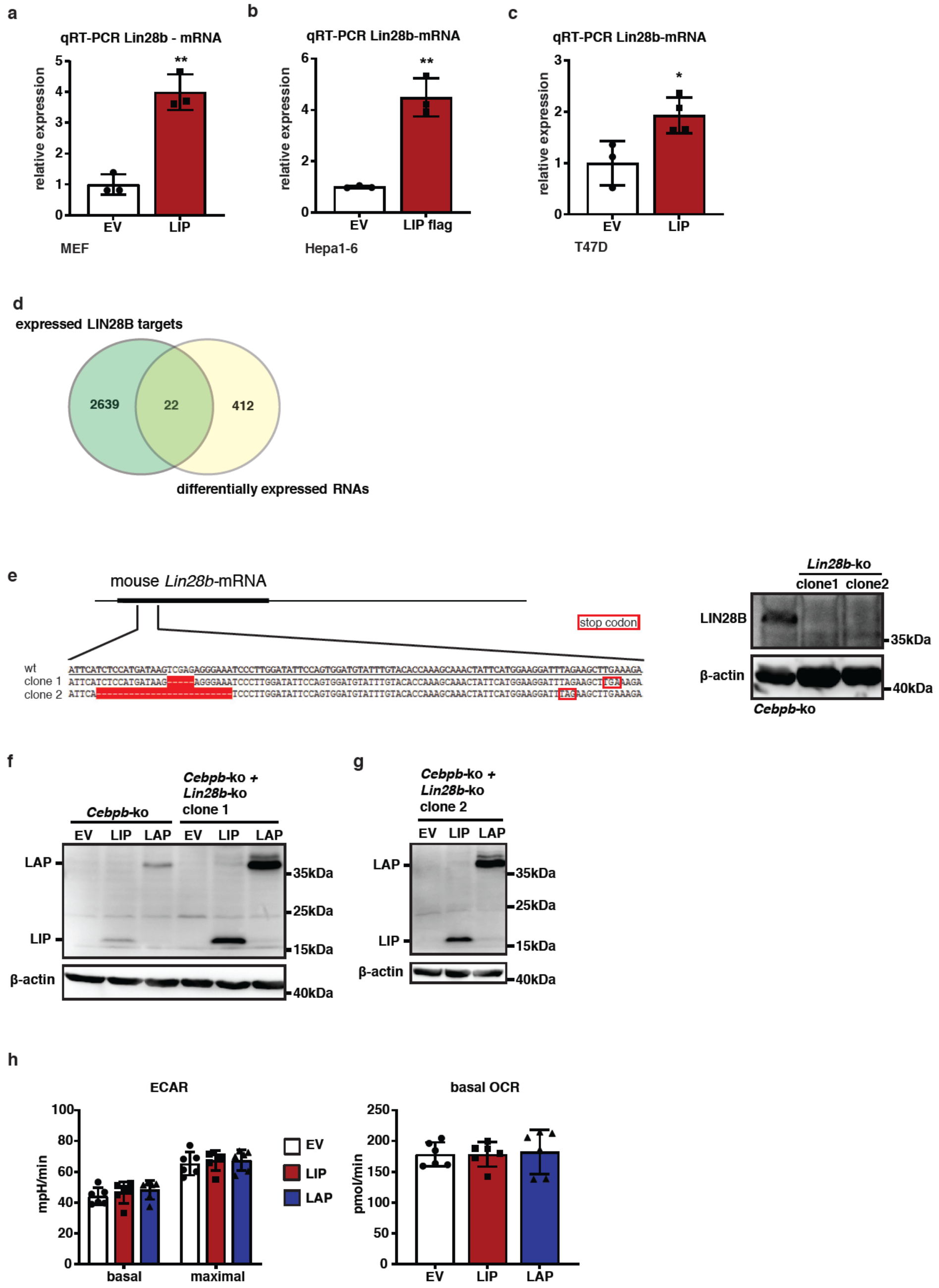

Supplementary Figure 3 LIP requires *Lin28b* to regulate cellular metabolism. **a** Relative *Lin28b*-mRNA expression levels by qRT-PCR (n=3) in wt MEFs expressing LIP compared to control (EV). **b** Relative *Lin28b*-mRNA expression levels by qRT-PCR (n=3) in Hepa1-6 cells expressing LIPflag compared to control (EV), **c** Relative *Lin28b*-mRNA expression levels by qRT-PCR (n=3) in T47D cells expressing LIP compared to control (EV). **d** Venn diagram showing overlap between LIP-regulated mRNAs (transcriptome) and LIN28B targets. **e** *Lin28b* sequence of clones 1 and 2 with CRISPR/Cas9 induced deletions in the LIN28B coding sequence that result in downstream in-frame stop codons. Immunoblot analysis at the right shows lack of LIN28B protein expression in both clones. Uncropped images are shown in Supplementary Fig. 7. **f** Left half of the immunoblot analysis shows LIP and LAP expression in *Cebpb*-ko MEFs, with ectopic expression of LIP, or LAP. Right half of the blot shows same MEFs with additional *Lin28b*-ko (clone1). Uncropped images are shown in Supplementary Fig. 7. **g** Same analysis as in G for clone 2. β-actin is used for loading control. Uncropped images are shown in Supplementary Fig. 7. **h** Basal ECAR and OCR of, C/EBPβ-KO MEFs (EV), *Cebpb*-ko MEFs with ectopic expression of LIP or LAP and additional *Lin28b*-ko for clone 2(n=6). Statistical differences were analyzed by Student's t-tests. Error bars represent ±SD, *P<0.05, **P<0.01, ***P<0. 001.

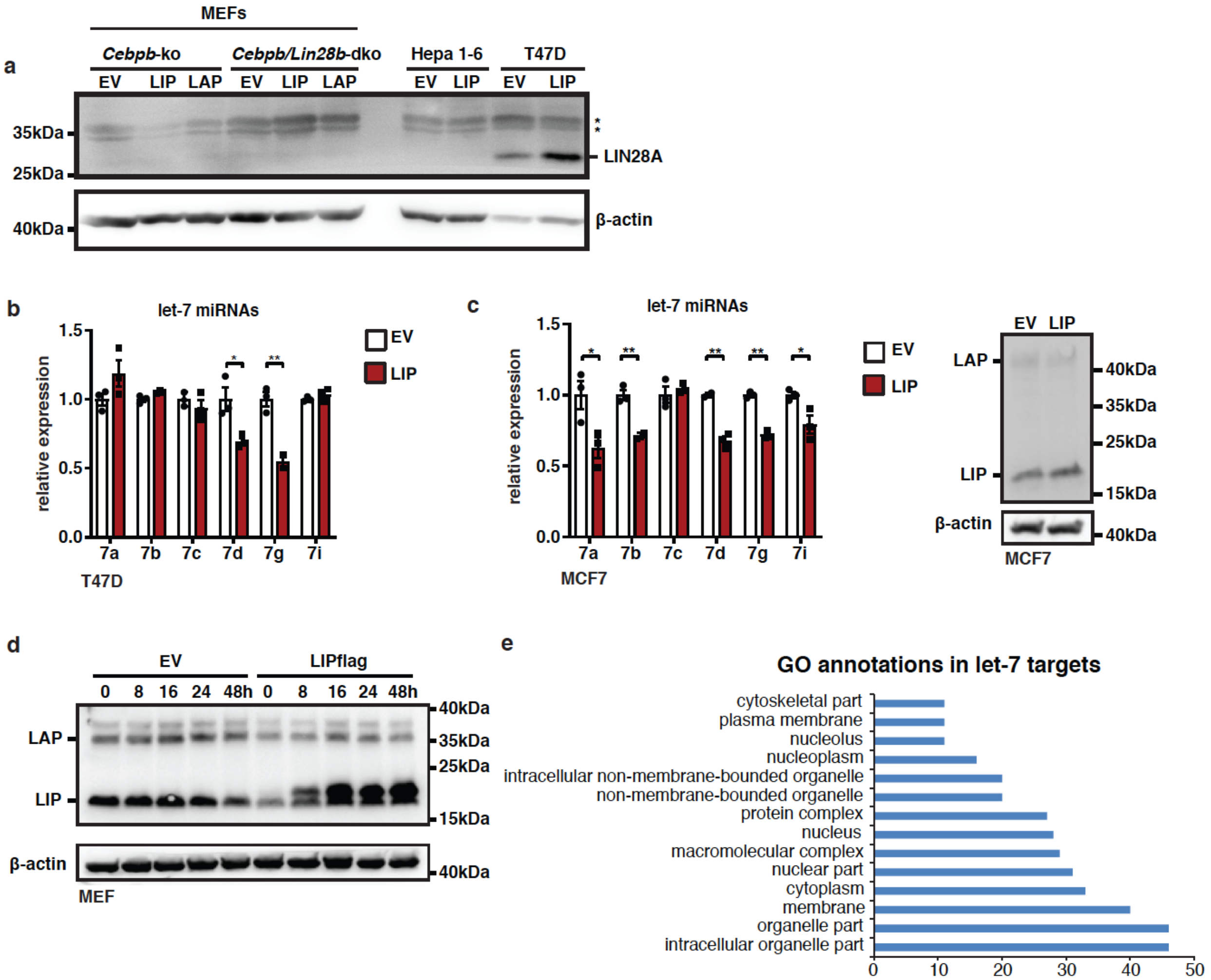

Supplementary Figure 4 LIP regulates *let-7* miRNAs. **a** Immunoblot analysis using a LIN28A specific antibody with extracts from different cell lines as indicated (bands marked with * represent unspecific signals). Uncropped images are shown in Supplementary Fig. 7. **b** Expression levels of let-7a, b, c, d, g and i in T47D cells ectopically expressing LIP compared to control (EV)(n=3). For LAP and LIP protein expression see immunoblot Figure S1g. **c** Expression levels of let-7a, b, c, d, g and i in MCF7 cells ectopically expressing LIP compared to control (EV)(n=3). Immunoblot analysis shows LAP and LIP protein expression and β-actin as loading control. Uncropped images are shown in Supplementary Fig. 7. **d** Immunoblot analysis of LAP and LIP expression in control MEFs (EV) and upon cumate-inducible expression of LIPflag using wt MEFs. β-actin is used for loading control. Uncropped images are shown in Supplementary Fig. 7. **e** (GOBP annotations of let-7 targets that are upregulated in the LIP proteome. Statistical differences were analyzed by Student's t-tests. Error bars represent ±SD, *P<0.05, **P<0.01.

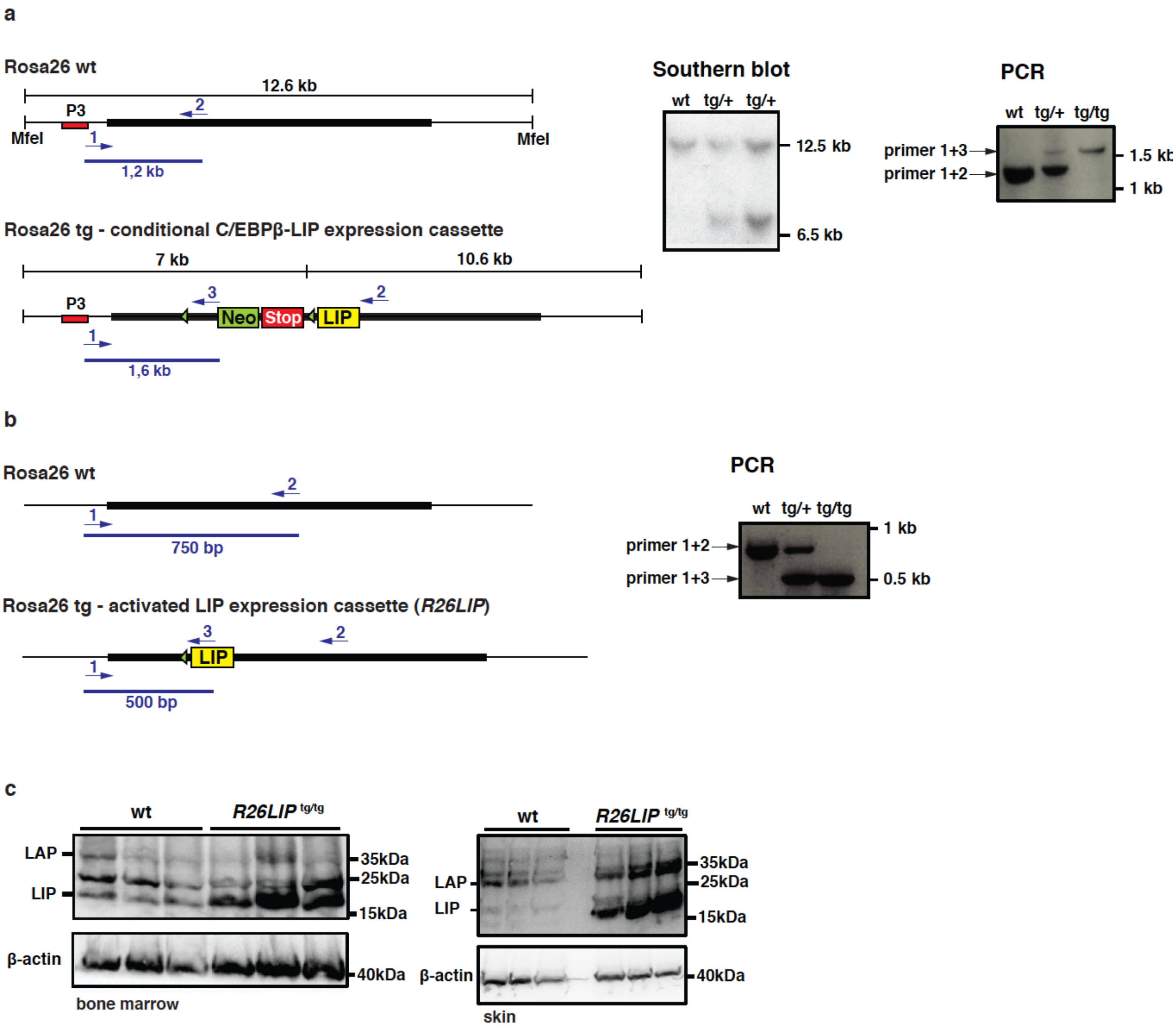

Supplementary Figure 5 LIP regulates let-7/LIN28B *in vivo*. **a** Schematic visualization of Rosa26 locus before and after adding an conditional LIP expression cassette. P3 marks the area where the southern blot probe binds and the arrows 1 to 3 mark the primers that are used for genotyping. Pictures of the southern blot film and genotyping PCR gel shows successful modification of the loci. **b** Schematic visualization of Rosa26 locus without and with the activated LIP expression cassette. The arrows 1 to 3 mark the primers that are used for genotyping. The genotyping PCR gel show successful modification of the loci. **c** Immunoblot analysis of LAP and LIP for wt and *R26LIP* mice of bone marrow, skin and spleen. Uncropped images are shown in Supplementary Fig. 7.

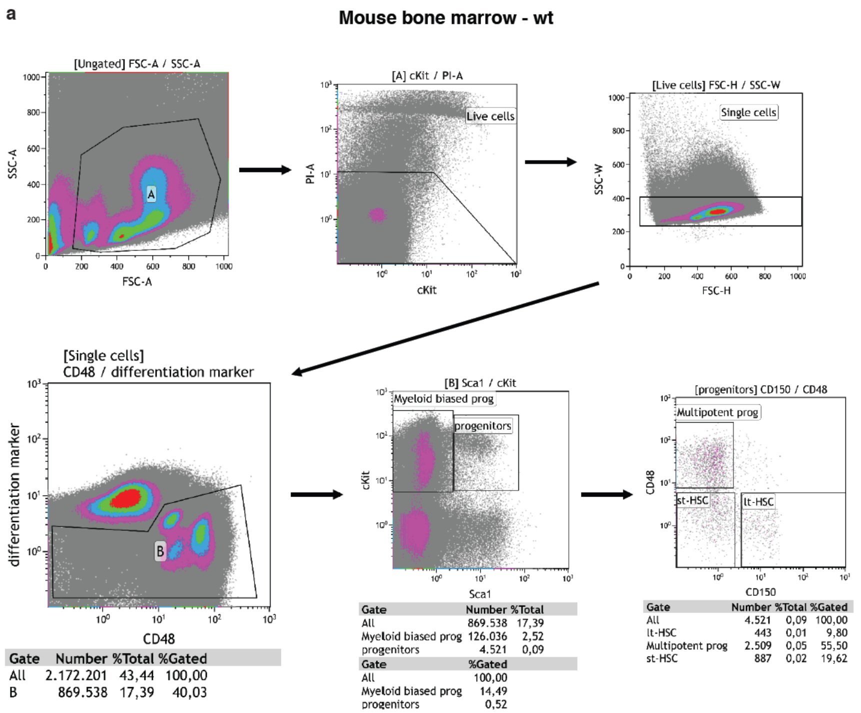

Gate	Number	%Total	%Gated
All	2.172.201	43,44	100,00
B	869.538	17,39	40,03

Gate	Number	%Total
All	869.538	17,39
Myeloid biased prog	126.036	2,52
progenitors	4.521	0,09

Gate	%Gated
All	100,00
Myeloid biased prog	14,49
progenitors	0,52

Gate	Number	%Total	%Gated
All	4.521	0,09	100,00
lt-HSC	443	0,01	9,80
Multipotent prog	2.509	0,05	55,50
st-HSC	887	0,02	19,62

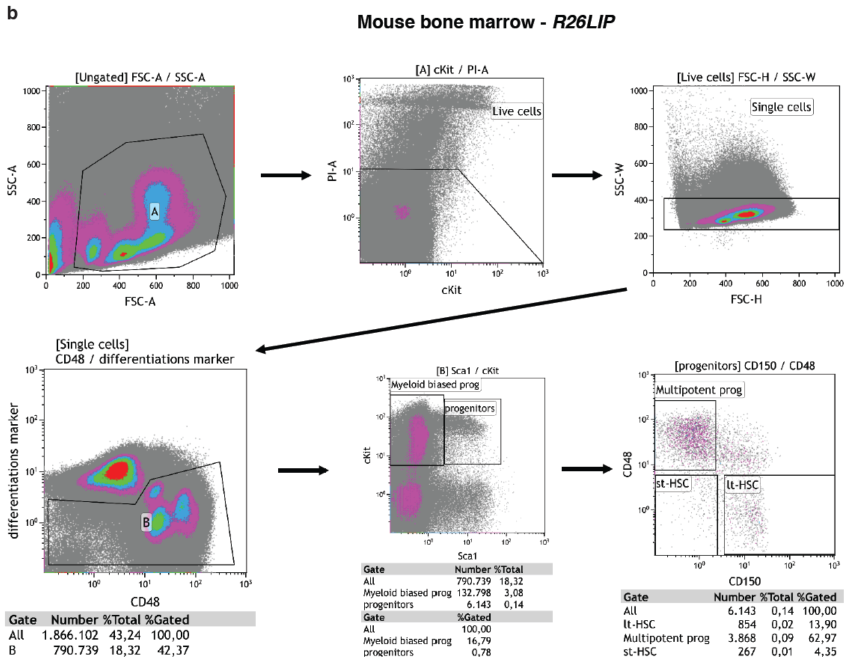

Gate	Number	%Total	%Gated
All	1.866.102	43,24	100,00
B	790.739	18,32	42,37

Gate	Number	%Total
All	790.739	18,32
Myeloid biased prog	132.798	3,08
progenitors	6.143	0,14

Gate	%Gated
All	100,00
Myeloid biased prog	16,79
progenitors	0,78

Gate	Number	%Total	%Gated
All	6.143	0,14	100,00
lt-HSC	854	0,02	13,90
Multipotent prog	3.868	0,09	62,97
st-HSC	267	0,01	4,35

Supplementary Figure 6 LIP increases progenitor frequency in mouse bone marrow. Gating schedule applied to flow cytometry data from wt (a) and *R26LIP* (b) mice. Cells were gated for forward scattering light (FSC) and side scattering light (SSC) abilities to identify particles/cells of a certain size. In the second graph cells/particles were selected for PI negativity and in the third for single cells. The fourth graph identifies Lin$^+$ and Lin$^-$ cells. The Lin$^-$ fraction was further analysed for c-Kit and Sca1 expression and the c-Kit$^+$/Sca1$^+$ positive fraction was further analysed for CD48 and CD150 expression to identify the fraction of long term (LT) hematopoietic stem cells (HSC), short term (ST) HSC and multipotent progenitors (MPP).

Supplementary Figure 7 Full scans of immunoblots. The areas used in the figures are boxed.

Figure 1

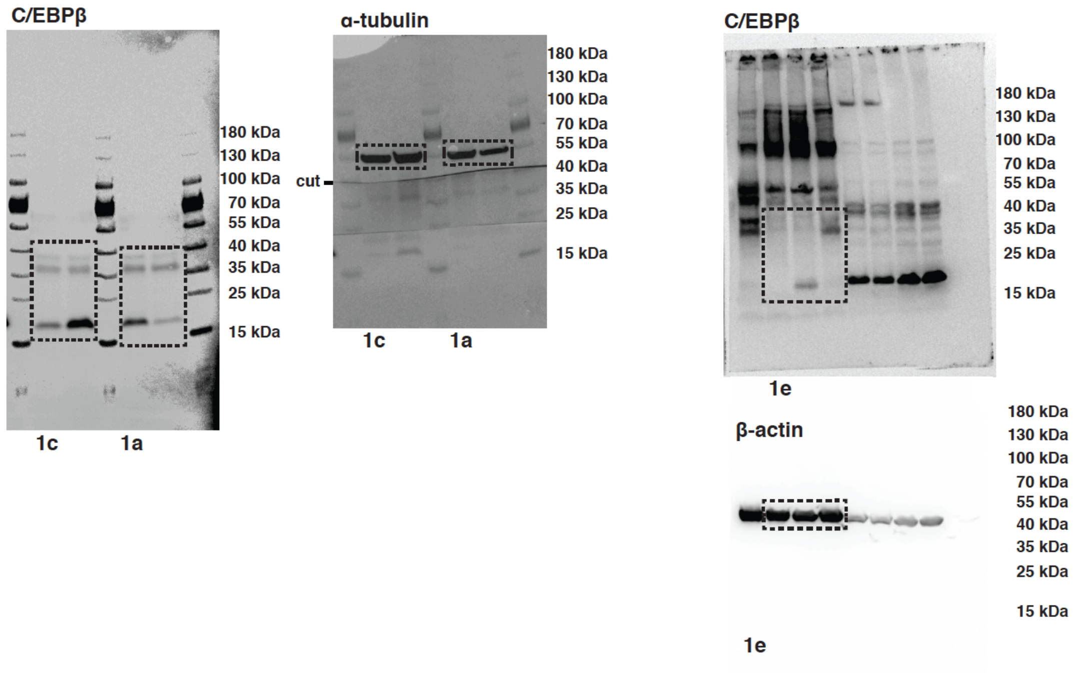

Figure 3

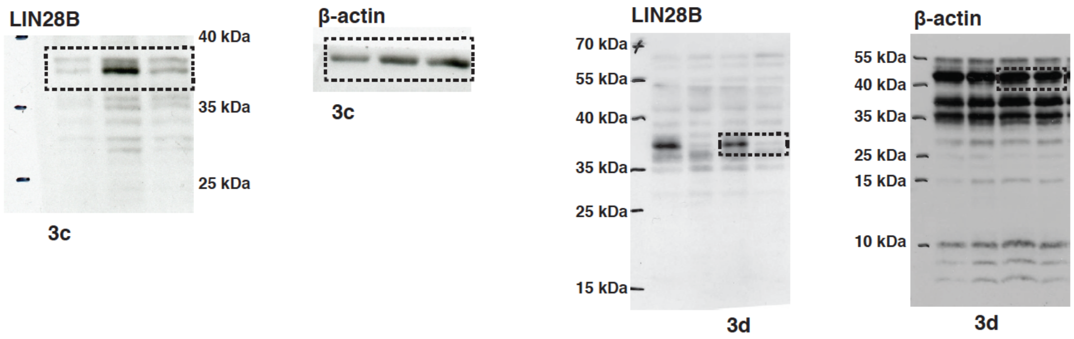

Supplementary Figure 1

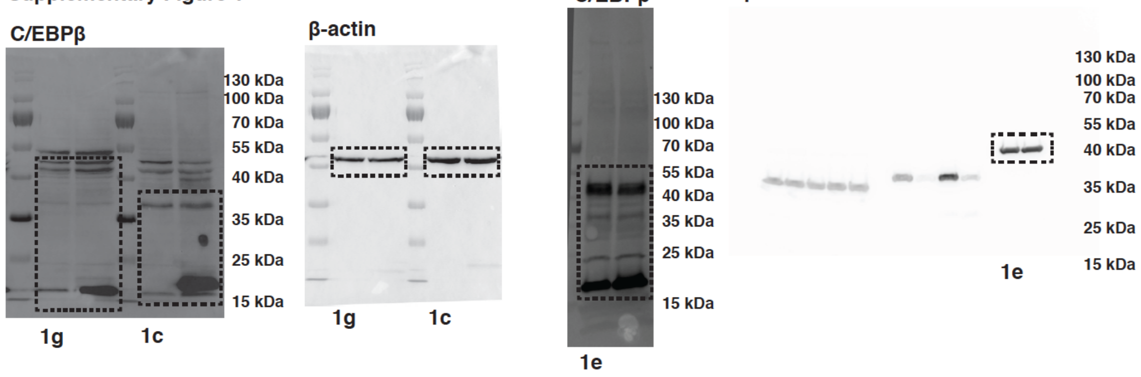

Supplementary Figure 3

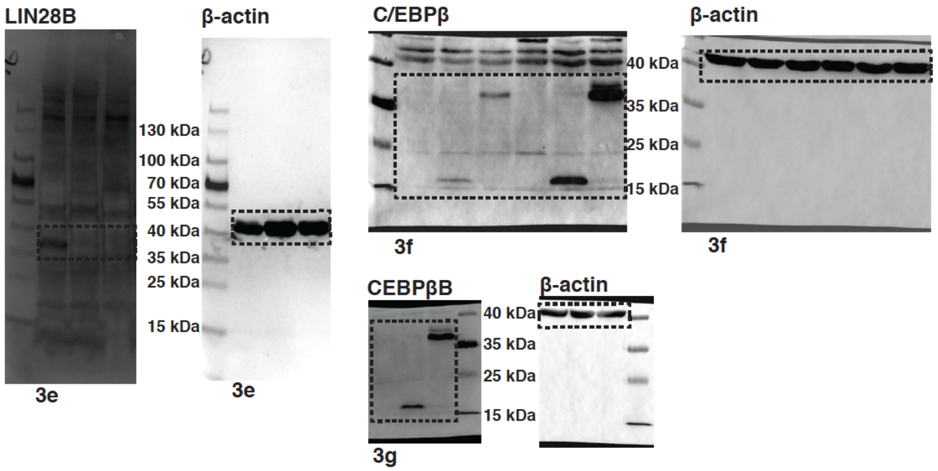

Supplementary Figure 4

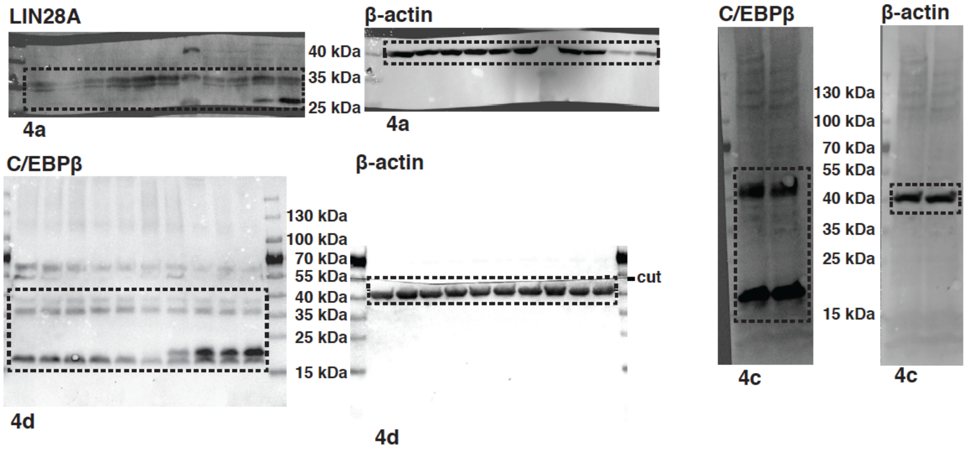

Supplementary Figure 5

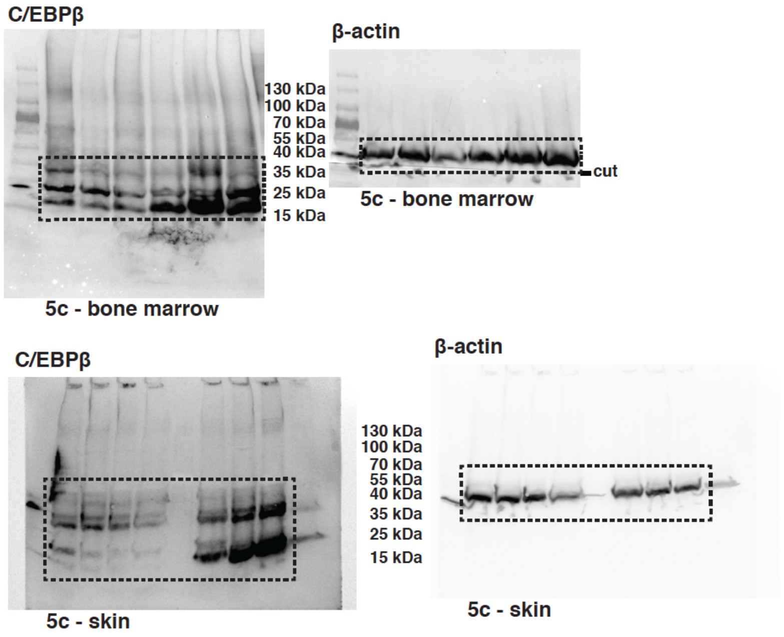

Supplementary Table 1 changes in mRNA or protein levels in MEFs overexpressing LIP or empty vector (EV) control

Pentose-phosphate pathway, related to Fig. 3e

mRNA	DESeq/EdgeR p-value	protein	t-test p-value
G6pdx	1	G6pdx	0.05
Pgd	1	Pgd	0.03
Taldo1	1	Pgls	0.85
Hibadh	1	Rpe	0.35
Rpe	1	Rpia	0.18
Glyr1	1		
H6pd	0.34		
Tpi1	1		
Pgls	1		

Glycolytic pathway, related to Fig. 3f

mRNA	DESeq/EdgeR p value	protein	t-test p value
Adpgk	1	Adpgk	0.10
Aldoa	1	Aldoa	<0.001
Aldoc	0.73	Eno1	0.42
Bpgm	0.44	Eno1	0.01
Dhtkd1	1	Eno2	0.42
Eno1	1	Eno3	0.73
Eno2	1	Gapdh	0.19
Eno3	1	Gpi1	0.13
Gapdh	1	Hk1	0.04
Gapdhs	1	Hk2	0.14
Gpi1	1	Ist1	0.05
Hk1	1	Ogdh	0.04
Hk2	1	Pdha1	0.02
Hkdc1	1	Pdha2	0.42
Ogdh	1	Pfkl	0.50
Pdha1	1	Pfkm	0.53
Pfkfb2	1	Pfkp	0.10
Pfkl	1	Pgam1	0.01
Pfkm	1	Pgk1	0.07
Pfkp	1	Pgk2	0.42
Pgam1	1	Pkm	0.04
Pgam2	1	Pklr	0.35
Pgk1	1	Tpi1	0.07
Tpi1	1		

p-values shown are calculated by combining results from DESeq and EdgeR analysis for the mRNAs or Student's t-tests for proteins.

Supplementary Table 2 C/EBPβ-associated DNA fragments

Cluster	location	C/EBPβ site position	Cell line	Score (out of 1000)	Rel. to start site/locus	associated with H3K27Ac
let7-a1/f1/d	intergenic	chr9:96929371-96929675	HeLa-S3, HepG2	308	-8559 bp	Yes
		chr9:96930928-96931214	HeLa-S3, IMR90, K562	438	-7020 bp	Yes
miR-100/ let-7a-2/miR125b-1	intergenic	chr11:122032890-122033184	A549, HeLa-S3, IMR90	422	-15660 bp	Yes
		chr11:122009483-122009738	IMR90	282	+7747 bp	Yes
		chr11:122007819-122008082	HeLa-S3, IMR90	330	+9148 bp	Yes
let-7a-3/b	intergenic	chr22:46481590-46481877	A549, HeLa-S3, K562	933	0	Yes
		chr22:46501859-46502137	A549, HeLa-S3, HepG2, IMR90	982	+19982 bp	Yes
miR-99a/ let-7c/miR125b-2	intronic? (C21orf34)	chr21:17892041-17892304	HeLa-S3	352	-19839 bp	No
		chr21:17894923-17895138	HeLa-S3, IMR90	878	-17220 bp	No
		chr21:17907438-17907551	HeLa-S3	555	-4592 bp	Yes
		chr21:17908980-17909243	HeLa-S3	326	-2900 bp	Yes
		chr21:17923366-17923518	HeLa-S3	654	+11130 bp	Yes
		chr21:17923911-17924062	HeLa-S3	983	+11675 bp	Yes
miR-99b/ let-7e/miR125a	intergenic	chr19:52183772-52184057	A549, H1-hESC, HepG2, IMR90, K562	975	-12267 bp	No
		chr19:52205637-52205912	K562	307	+9598 bp	Yes
		chr19:52216011-52216316	A549, H1-hESC, K562	243	+19972 bp	No
let-7f-2/miR-98	intronic (HUWE1)	chrX:53709195-53709458	HeLa-S3	196	+4215 bp	Yes
let-7g	intronic (WDR82)	chr3:52312731-52312994	HeLa-S3	200	-72 bp	Yes
let-7i	intergenic	chr12:62996121-62996415	A549, HeLa-S3, IMR90, K562	482	-1051 bp	Yes
		chr12:62997376-62997650	A549, HeLa-S3, HepG2	260	+184 bp	Yes

Data retrieved from http://genome.ucsc.edu/ENCODE/

Supplementary Table 3 Skin phenotypes, related to Fig. 7a

ID	Genotype	Skin phenotype	Predominant hair follicular type
wt 1	wt	Normal	Telogen
wt 2	wt	Normal	Anagen
wt 3	wt	Normal	Telogen
wt 4	wt	Normal	Telogen
wt 5	wt	Normal	Telogen
wt 6	wt	Normal	Telogen/Anagen
R26LIP 1	R26LIP	Mild patchy epidermal hyperplasia (score 1)	Telogen
R26LIP 2	R26LIP	Mild patchy epidermal hyperplasia (score 0.5)	Telogen/Anagen
R26LIP 3	R26LIP	Moderate epidermal hyperplasia (score 2.5)	Anagen
R26LIP 4	R26LIP	Normal	Telogen/Anagen
R26LIP 5	R26LIP	Mild patchy epidermal hyperplasia (score 1)	Telogen/Anagen
R26LIP 6	R26LIP	Mild patchy epidermal hyperplasia (score 0.5)	Telogen

Supplementary Table 4 Primer sequences or ordering information in case sequence is not publicly available.

Target	Primer 1 forward	Primer 2 reversed	Figure	Suppl. Figure
Cytochrome b	CATTTATTATCGCGGCCC	TGTTGGGTTGTTTGATCCTG	1g	
β-actin	AGAGGGAAATCGTGCGTGAC	CAATAGTGATGACCTGGCCGT	1g	
let-7 primers	let-7 isoform specific primer miScript II RT kit, Qiagen	miScript Universal Primer	5a, b, c, f, 6a, 7o	4b, c
let-7a	UGAGGUAGUAGGUUGUAUAGUU	miScript Universal Primer		
let-7b	UGAGGUAGUAGGUUGUGUGGUU	miScript Universal Primer		
let-7c	UGAGGUAGUAGGUUGUAU**G**GUU	miScript Universal Primer		
let-7d	**A**GAGGUAGUAGGUUG**C**AUAGUU	miScript Universal Primer		
let-7e	UGAGGUAGGAGGUUGUAUAGUU	miScript Universal Primer		
let-7f	UGAGGUAGUAGAUUGUAUAGUU	miScript Universal Primer		
let-7g	UGAGGUAGUAG**U**UUGUA**C**AGUU	miScript Universal Primer		
let-7i	UGAGGUAGUAGUUUGUGCUGUU	miScript Universal Primer		

Snord72	Hs_SNORD72_11 miScript Primer Assay (MS00033719), Qiagen	5a, b, c, f, 6a, 7o	4b, c
U6	Hs_RNU6-2_11 miScript Primer Assay (MS00033740), Qiagen	5c	
Lin28b human	RT² qPCR Primer Assay for Human LIN28B (PPH57843B), Qiagen		3c
Lin28b mouse	RT² qPCR Primer Assay for Mouse Lin28b (PPM31163B), Qiagen	4b, d, 5g, 6b	3a,b
