# Supplementary material for: C/EBPβ-LIP induces cancer-type metabolic reprogramming by regulating the let-7/LIN28B circuit in mice
Source: Commun Biol. 2019 Jun 14;2:208. doi: 10.1038/s42003-019-0461-z (PMC6572810; doi:10.1038/s42003-019-0461-z)
Supplement: Supplementary file 3 — Reporting Summary [file 42003_2019_461_MOESM3_ESM.pdf]

nature research

Corresponding author(s): Cornelis F. Calkhoven

Last updated by author(s): Apr 24, 2019

Reporting Summary

Nature Research wishes to improve the reproducibility of the work that we publish. This form provides structure for consistency and transparency in reporting. For further information on Nature Research policies, see Authors & Referees and the Editorial Policy Checklist.

Statistics

For all statistical analyses, confirm that the following items are present in the figure legend, table legend, main text, or Methods section.

n/a	Confirmed	
☐	☒	The exact sample size (*n*) for each experimental group/condition, given as a discrete number and unit of measurement
☐	☒	A statement on whether measurements were taken from distinct samples or whether the same sample was measured repeatedly
☐	☒	The statistical test(s) used AND whether they are one- or two-sided<br>*Only common tests should be described solely by name; describe more complex techniques in the Methods section.*
☐	☒	A description of all covariates tested
☐	☒	A description of any assumptions or corrections, such as tests of normality and adjustment for multiple comparisons
☐	☒	A full description of the statistical parameters including central tendency (e.g. means) or other basic estimates (e.g. regression coefficient) AND variation (e.g. standard deviation) or associated estimates of uncertainty (e.g. confidence intervals)
☐	☒	For null hypothesis testing, the test statistic (e.g. *F*, *t*, *r*) with confidence intervals, effect sizes, degrees of freedom and *P* value noted<br>*Give P values as exact values whenever suitable.*
☒	☐	For Bayesian analysis, information on the choice of priors and Markov chain Monte Carlo settings
☒	☐	For hierarchical and complex designs, identification of the appropriate level for tests and full reporting of outcomes
☒	☐	Estimates of effect sizes (e.g. Cohen's *d*, Pearson's *r*), indicating how they were calculated

Our web collection on statistics for biologists contains articles on many of the points above.

Software and code

Policy information about availability of computer code

Data collection	Microsoft Excel 2010, GraphPad Prism 6, Roche LightCycler® 480 Software, ImageQuant TL 8.1, R, PERSEUS, MaxQuant proteomics pipeline (v1.5.3.30), TissueFAXS Imaging Software, ImageJ, Wave Desktop
Data analysis	two tailed t-test, edgeR and DESeq packages for R

For manuscripts utilizing custom algorithms or software that are central to the research but not yet described in published literature, software must be made available to editors/reviewers. We strongly encourage code deposition in a community repository (e.g. GitHub). See the Nature Research guidelines for submitting code & software for further information.

Data

Policy information about availability of data

All manuscripts must include a data availability statement. This statement should provide the following information, where applicable:

- Accession codes, unique identifiers, or web links for publicly available datasets
- A list of figures that have associated raw data
- A description of any restrictions on data availability

Data availability
RNA-sequencing data as presented in Fig. 3 and Supplementary Fig. 2 are available at NCBI's gene expression omnibus (GEO, https://www.ncbi.nlm.nih.gov/geo/)61 with identifier GSE110316. The mass spectrometry proteomics data as presented in Fig. 3 have been deposited to the ProteomeXchange (http://www.proteomexchange.org) Consortium via the PRIDE62,63 partner repository with the dataset identifier PXD010095.

October 2018

Field-specific reporting

Please select the one below that is the best fit for your research. If you are not sure, read the appropriate sections before making your selection.

☒ Life sciences ☐ Behavioural & social sciences ☐ Ecological, evolutionary & environmental sciences

For a reference copy of the document with all sections, see nature.com/documents/nr-reporting-summary-flat.pdf

Life sciences study design

All studies must disclose on these points even when the disclosure is negative.

Sample size	No statistical method was used to determine the sample size. Most sample sizes were picked to allow statistical analysis.
Data exclusions	No data was excluded.
Replication	Most cell culture experiments were reproduced three times as specified in the figure legends. The mouse experiments were done once with animals of different litters.
Randomization	Randomization is not relevant to this study because effects of protein overexpression or down regulation were investigated. Therefore different cell lines had to be used. However, upon generation of the cell lines cells were not selected for special features.
Blinding	Investigators were not blinded to group allocation during the study. This was not possible since for most experiments the person who performed the experiment, collected the data and analyzed the data was the same.

Reporting for specific materials, systems and methods

We require information from authors about some types of materials, experimental systems and methods used in many studies. Here, indicate whether each material, system or method listed is relevant to your study. If you are not sure if a list item applies to your research, read the appropriate section before selecting a response.

Materials & experimental systems

n/a	Involved in the study	
☐	☒	Antibodies
☐	☒	Eukaryotic cell lines
☒	☐	Palaeontology
☐	☒	Animals and other organisms
☒	☐	Human research participants
☒	☐	Clinical data

Methods

n/a	Involved in the study	
☒	☐	ChIP-seq
☒	☐	Flow cytometry
☒	☐	MRI-based neuroimaging

Antibodies

Antibodies used	C/EBPβ (E299) from Abcam, LIN28B (mouse preferred) from Cell Signaling Technology, β-tubulin (GT114) from GeneTex and β-actin (clone C4) (#691001) from MP Biomedicals.
Validation	C/EBPβ (E299): Figure 1E shows that C/EBPβ signal is absent in C/EBPβ knock out fibroblast and C/EBPβ knock out fibroblast with overexpression of LIP or LAP show specific signals for the single isoforms.<br>LIN28B (mouse preferred): Figure S3E shows that Lin28b signal is absent in Lin28b knock out cells.

Eukaryotic cell lines

Policy information about cell lines

Cell line source(s)	ATCC
Authentication	By microscopic morphology
Mycoplasma contamination	The cell lines were not tested for mycoplasma contamination.
Commonly misidentified lines<br>(See ICLAC register)	none

Animals and other organisms

Policy information about studies involving animals; ARRIVE guidelines recommended for reporting animal research

Laboratory animals	Details of animal experiments and licence can be found in the manuscript's material and methods section.
Wild animals	not used
Field-collected samples	not used
Ethics oversight	All of the animals were handled according to approved institutional animal care and use committee (IACUC) protocols of the University of Groningen (#6996A).

Note that full information on the approval of the study protocol must also be provided in the manuscript.
